# Prioritizing meaning: value, prediction, and age differences in sentence processing

**DOI:** 10.3389/fcogn.2026.1862899

**Published:** 2026-10-08

**Authors:** Yu Min W. Chung, Kara D. Federmeier

**Affiliations:** 1 Department of Psychology, University of Illinois Urbana–Champaign, Champaign, IL, United States; 2 Beckman Institute for Advanced Science and Technology, University of Illinois Urbana–Champaign, Champaign, IL, United States

**Keywords:** aging, event-related potentials (ERPs), predictive processing, sentence processing, value-directed remembering

## Abstract

**Introduction:**

Across three experiments, we investigated how people process important but complex information and how this may change with age.

**Methods:**

Using an adaptation of the value-directed remembering (VDR) paradigm for sentences that varied in constraint and predictability, younger and older adults read sentences associated with high or low value for subsequent memory tests. Behavioral measures of recall and recognition were collected, and event-related potentials (ERPs) were recorded during reading to examine the processing strategies engaged during sentence comprehension.

**Results:**

Behaviorally, both younger and older adults used value information to prioritize memory, showing better recall for high- than low-value sentences, alongside patterns consistent with prediction-based memory distortions (e.g., increased false recognition for predictable but unseen items). At the neural level, across age groups, a post-N400 anterior positivity elicited by unexpected words in strongly constraining sentences, a pattern associated with revising disconfirmed predictions, was not modulated by value. In contrast, value reliably influenced a late frontal negativity elicited by moderately to strongly expected words and thought to reflect message-level integration, with larger effects for high-value sentences. Younger adults also showed enhanced N400 expectancy effects for high-value items, indicating increased semantic facilitation, whereas older adults did not show value-based modulation of the N400. Sorting ERPs by subsequent recall revealed little evidence that sentence-final word processing robustly predicted later memory in either group.

**Discussion:**

Together, these findings suggest that both younger and older adults use value information to prioritize processing, with particularly clear evidence for enhanced message-level integration. At the same time, sensitivity to prediction violations, and the predictive processes that give rise to them, appeared relatively insensitive to value, consistent with the idea that predictive processing operates in a state-like manner. Notably, with sufficient task support, older adults showed evidence of engaging prediction-related processes typically reduced in aging, although this engagement did not extend to modulation of earlier semantic facilitation and did not fully eliminate age-related differences in comprehension.

## 1 Introduction

People are routinely confronted with more information than they can retain in memory, requiring them to prioritize what is most important to remember. For example, recalling the name of a medication that has just been prescribed would likely be more important than remembering the name of the receptionist who greeted you at your appointment. One well-established approach to studying people's ability to selectively retain important information is through the value-directed remembering (VDR) paradigm (Castel et al., 2002; Watkins and Bloom, 1999). In this paradigm, participants study a set of stimuli, typically relatively simple materials such as single words (Castel et al., 2002), visual scenes (Adcock et al., 2006), or proper names (Festini et al., 2013), each of which is paired with a point value representing the reward for recalling it, with higher values indicating more important information. Studies using this paradigm have shown that people are able to selectively encode and recall higher-value items, demonstrating that memory is shaped not only by capacity limits but also by both automatic and strategic processes that prioritize important content (Knowlton and Castel, 2022).

### 1.1 Value-directed remembering and aging

One key question concerns whether and, if so, how the ability to prioritize information changes with aging. Although aging is associated with declines in many aspects of episodic memory, older adults have been shown to retain the ability to successfully use value information to guide memory for a variety of stimuli, including words in lists (Castel et al., 2011), grocery prices (Castel et al., 2013), allergens (Middlebrooks et al., 2016), and medication side effects (Friedman et al., 2015). At the same time, the implementation of value-based prioritization can differ with age. For example, older adults sometimes show reduced selectivity early in learning or require more experience with a task to adopt effective value-based strategies (Castel et al., 2002). More generally, value-based selectivity appears to depend on a combination of motivational and strategic factors. For example, understanding the value structure of a task seems critical for allowing both younger and older adults to strategically allocate attention and encoding effort toward high-value information (Schwartz et al., 2023). At the same time, the effectiveness of value cues can depend on task demands such as time pressure, interference, and the strategic opportunities afforded by the task (Miller and Castel, 2025; Murphy et al., 2023), and older adults may be more affected (e.g., Hoover et al., 2024). These findings suggest that value-based prioritization relies in part on strategic control processes, raising the possibility that differences in how younger and older adults deploy such strategies could influence how effectively value cues guide memory.

One way task structure may shape these opportunities is through the timing of value information. In many VDR studies, stimuli are presented as discrete items and value information is presented concurrently with the to-be-remembered information (e.g., Castel et al., 2011; Murphy et al., 2023). However, even for relatively simple stimuli, work manipulating the timing of value cues suggests that providing value information before encoding can be especially advantageous (e.g., Soderstrom and McCabe, 2011; Villaseñor et al., 2021, cf. Hayes et al., 2013). When value is known in advance, participants can allocate cognitive resources more strategically, prioritizing high-value information during encoding (e.g., Ariel et al., 2009), for example by devoting additional time or deeper processing to stimuli that they know in advance are important (e.g., Ariel et al., 2015; Cohen et al., 2017). Beyond these kinds of general memory control strategies, more complex materials, such as sentences, invite additional kinds of processing strategies that may be influenced by value cues. As reviewed in Federmeier (2022), sentence comprehension can proceed through the use of relatively passive mechanisms but can also engage more active strategies, including prediction. Active prediction not only shapes online word processing but also has important consequences for people's downstream memory of what they have comprehended (e.g., Rommers and Federmeier, 2018a,b; Hubbard et al., 2019; Hubbard and Federmeier, 2024). This raises the question of whether people adapt their language comprehension strategies under different value conditions, and, if so, whether these adaptations are effective and used similarly by older and younger adults.

### 1.2 Value cues during sentence comprehension

Extensive research indicates that aging is associated with qualitative changes in language processing (Wlotko et al., 2010), particularly in how sentence context information is used during comprehension (see reviews by Payne and Silcox, 2019; Stine-Morrow and Radvansky, 2018). On the one hand, older adults often seem to rely more on prior linguistic context, particularly when bottom-up information is noisy or degraded (Schneider et al., 2000; Speranza et al., 2000), both for single sentences (Choi et al., 2017) and for larger text (Miller and Stine-Morrow, 1998; Stine-Morrow et al., 1996). However, older adults may use sentence context information differently from younger adults. They often appear to rely less routinely on active prediction (Cheimariou et al., 2021; Federmeier et al., 2010; Wlotko et al., 2012; reviewed in Federmeier et al., 2026), but may engage more strongly in later integrative processes (Stine-Morrow et al., 2001, 2010). Thus, the timing of value information may be particularly important for understanding how aging shapes the prioritization of linguistic information.

More generally, using value cues to prioritize linguistic information is likely to draw on domain-general mechanisms that support the allocation and regulation of processing resources during sentence encoding. EEG/ERP work with older adults suggests that such mechanisms can shape both online comprehension and memory. For example, cognitive control predicts older adults' flexible modulation of N400 predictability effects based on speaker reliability (Dave et al., 2021), and oscillatory work has linked age differences in sentence encoding success to alpha-band dynamics that may reflect inhibition/disinhibition during comprehension (Beese et al., 2019). Thus, age differences in value-based sentence processing may reflect not only differences in predictive or integrative language mechanisms *per se*, but also differences in the flexibility with which attention and control processes are recruited as sentence meaning unfolds.

Taken together, these findings suggest that value cues, especially when available in advance, could influence how language is processed during comprehension and that such effects might differ across age groups. As an initial step toward understanding how value cues interact with sentence processing, Chung and Federmeier (2023) adapted the VDR paradigm to examine memory for sentences in young adults. To capture effects related to active prediction, they used sentences that varied in how strongly they constrained for a particular sentence-final word (i.e., how readily they afforded a prediction) and in whether the final word was the predicted completion or an unexpected but plausible alternative. Value information was provided for each sentence, and, critically, the timing of the value cues was manipulated: cues appeared either before or after the sentence, which was presented word-by-word using rapid serial visual presentation (RSVP). Participants were asked both to recall the sentences and to complete a recognition test on the sentence-final words.

The results showed clear effects of value on sentence memory overall, as recall was enhanced for high-value sentences relative to low-value ones across conditions, demonstrating that, similar to patterns with simpler materials, young adult participants could effectively use value cues to prioritize memory for sentences. Importantly, the timing of the value cues further modulated memory patterns. When value information was provided in advance, recall of high-value sentences was poorer if those sentences ended with an unexpected word, suggesting that violated predictions disrupted encoding. At the same time, participants in this condition were more likely to falsely recognize (lure to) the predicted word from a strongly constraining context that had been read with an unexpected ending. These patterns are striking because they occurred for exactly the same materials, with the only difference being when value information was available and, correspondingly, what encoding strategies participants could use.

The fact that memory was sometimes better when value cues followed the sentence suggests that advance knowledge of importance may prompt comprehension strategies that do not always align with accurate memory. In particular, Chung and Federmeier (2023) argued that the tendency to misremember seeing predictable words and the negative impact of unexpected information on sentence recall both point to the possibility that young participants engage predictive processing to prioritize high value information when value cues are available in advance. However, because that study relied solely on memory outcomes, the cognitive processes engaged during sentence comprehension could only be inferred indirectly. To address this question, the present research examined how value cues influence the neural processes engaged during sentence comprehension, while also asking how these effects might differ in older adults.

### 1.3 Neural measures of sentence comprehension processes

Event-related potentials (ERPs) provide temporally precise indices of the processes that support language comprehension and have been widely used to characterize how contextual information and predictions influence word processing in both younger and older adults. Three ERP components are particularly relevant for examining these possibilities. The first is the N400, a negative-going potential that peaks around 400 ms after stimulus onset and has been closely tied to semantic access (Kutas and Federmeier, 2011; Federmeier, 2022). The N400 is elicited by all meaningful stimuli but its amplitude is reduced (i.e., becomes more positive) when less new semantic information must be activated – for example when features of an incoming word have already been activated by contextual information. Such contextual facilitation does not require active prediction but can be shaped by it. For instance, when sentences strongly constrain toward a particular completion, younger adults show N400 facilitation even for anomalous words that share semantic features with the predicted ending (“related anomaly effects;” Federmeier and Kutas, 1999; Federmeier et al., 2001), provided that readers have sufficient time to generate predictions (Wlotko and Federmeier, 2015). Consistent with the role of predictive engagement, the magnitude of N400 expectancy effects can also increase when readers are explicitly encouraged to generate predictions (Lai et al., 2024). In contrast, although older adults show robust N400 context effects overall (reviewed in Wlotko et al., 2010), they do not show the prediction-linked related anomaly effects (Federmeier and Kutas, 2019) or anticipatory effects on pre-target articles linked to predicted upcoming nouns (DeLong et al., 2012).

The N400 is followed by two components, the anterior positivity and the frontal negativity, which have been linked, respectively, to processes associated with prediction violation and message-level integration. The post-N400 anterior positivity (AP) is a positive-going component typically observed between approximately 600–900 ms after word onset over anterior scalp sites (Federmeier et al., 2007; Otten and Van Berkum, 2008; Payne and Federmeier, 2017a; Wlotko et al., 2012). This component is observed for unexpected but plausible completions of strongly constraining sentences, for which language comprehenders can generate predictions (Federmeier et al., 2007; Otten and Van Berkum, 2008; Payne and Federmeier, 2017a; Wlotko et al., 2012). Because it is not observed for implausible input (DeLong et al., 2014; Federmeier et al., 2010), the AP is thought to reflect processes involved in overriding or revising a prediction in order to incorporate new information into the developing sentence representation. Importantly, this prediction-violation AP effect has often been found to be reduced or absent in older adults (Wlotko et al., 2012), even in simplified contexts such as category-exemplar pairings (Federmeier et al., 2010). At the same time, some work suggests that the extent to which older adults engage predictive processing may depend on factors such as moment-by-moment processing dynamics (Jongman et al., 2023), individual differences in cognitive abilities (Federmeier et al., 2010), and the availability of task support (Dave et al., 2018).

Another ERP pattern observed in a similar time frame, also over frontal electrode sites, is the frontal negativity (FN). It is elicited by expected completions of moderately to strongly constraining sentences, particularly those that have a salient alternative ending, and has been linked to deeper message-level processing, such as resolving competition among alternative interpretations or refining aspects of the evolving sentence representation (Lee and Federmeier, 2009; Wlotko and Federmeier, 2012b). Importantly, this component appears to increase when readers engage more actively with the meaning of a sentence (Lai et al., 2024), suggesting that it may provide a useful index of the degree to which comprehension strategies are engaged. Different from the AP, the FN has been found to be intact, and possibly even augmented, in older adulthood (Wlotko and Federmeier, 2012a).

Thus, although the AP and FN are both late anterior effects measured over the same prefrontal channels and time window in the present study, they are distinguished by the contrasts used to isolate them and by their functional interpretation. Together, they provide complementary indices of how contextual information and predictions shape sentence processing. In particular, they allow us to examine whether value cues influence the degree to which readers engage predictive processing (AP) or instead rely on later integrative mechanisms (FN), and whether these patterns differ between younger and older adults. Therefore, in the current study we extended the work of Chung and Federmeier (2023) through three experiments designed to examine the cognitive processes engaged when young and older adults prioritize high-value sentences.

### 1.4 The present research

In Experiment 1, we recorded event-related potentials (ERPs) from younger adults as they completed the VDR task adapted to sentences by Chung and Federmeier (2023). We focused on the condition in which value cues were presented before the sentence, so that we would be able to assess how value information influences neural responses elicited during sentence encoding. The design and stimuli were thus identical to the Before condition of Chung and Federmeier (2023). Sentences varied in contextual constraint and ended with either expected or unexpected words, and each sentence was paired with either a low or high value presented before the sentence. We focused on ERP components associated with semantic facilitation (N400), prediction-related processing (AP), and message-level integration (FN) and examined responses to the sentence-final word as a function of value.

Prior work using these materials has shown graded N400 responses (smallest to expected completions in strongly constraining contexts, intermediate to expected items in weakly constraining contexts, and largest to unexpected items regardless of constraint; Federmeier et al., 2007; Wlotko et al., 2012). Younger adults also show an AP to unexpected endings in strongly constraining contexts, reflecting prediction violation (Federmeier et al., 2007; Wlotko et al., 2012; Hubbard and Federmeier, 2024). If participants use value cues to increase predictive engagement, one might expect a larger AP for high- than low-value sentences. However, recent work suggests that prediction may operate more like a state that is either engaged or not rather than a process graded by attention or effort (Hubbard and Federmeier, 2021; Lai et al., 2024). In that case, the AP might be absent for low-value items but present for high-value ones, or it may remain similar across conditions. Lai et al. (2024) further found that greater engagement in comprehension enhanced N400 facilitation to expected words and increased the FN effect. Accordingly, if value cues enhance message integration processes, we might observe that N400 expectancy effects and FN effects are larger in high compared to low value sentences. Finally, we use the recall data to backsort the ERPs, in order to try to identify processes that might lead to successful downstream recall of the sentences.

Experiment 2 then shifted the focus to older adults, first asking whether they show value-based prioritization of sentence memory and whether this prioritization depends on when value information becomes available. In particular, we sought to replicate the behavioral findings of Chung and Federmeier (2023), testing whether value cues presented before vs. after the sentence differentially shape memory in aging. This manipulation allowed us to ask whether older adults' memory for the sentences and the sentence-ending words benefits more when value is available during sentence encoding, where it can guide processing as the sentence unfolds, or after sentence presentation, where it can influence later rehearsal, evaluation, or memory strategies. If older adults rely less on moment-by-moment predictive processing and more on later integrative “wrap-up” processes (e.g., Stine-Morrow et al., 2001, 2010), they may benefit comparatively more from value information presented after the sentence. Moreover, because older adults often engage predictive mechanisms less consistently (Federmeier et al., 2026), they may be less likely to selectively adjust strategies for high- vs. low-value sentences when value is provided in advance. Alternatively, if older adults use value cues to guide strategic processing in a manner similar to younger adults, the distribution of memory outcomes in the Before condition should resemble the pattern observed in Chung and Federmeier (2023), including increased recognition of unexpected endings and reduced recall for high-value sentences that ended unpredictably.

Finally, Experiment 3 asked how the online processes older adults engage during sentence comprehension are influenced when value information is available in advance to shape those processes. To this end, we recorded ERPs from older adults, as in Experiment 1 focusing on the condition in which value cues are provided before each sentence. A central question was whether value cues might lead older adults to engage predictive processing even though they do not routinely rely on such mechanisms during sentence comprehension. If so, unexpected endings might elicit AP patterns similar to those observed in young adults. At the same time, value cues might influence comprehension in other ways. Rather than modulating prediction, they may instead enhance semantic facilitation for expected words, leading to larger N400 reductions, or promote deeper engagement with sentence meaning, reflected in increased FN effects (e.g., Lai et al., 2024). Accordingly, we again examined the N400, AP, and FN as potential indices of how value cues influence comprehension strategies in older adults, and we backsorted the ERPs based on the recall data.

## 2 Experiment 1

Experiment 1 examined whether value cues influence the engagement of sentence-processing strategies during comprehension by young adults. ERPs were recorded while participants completed the VDR task adapted from Chung and Federmeier (2023), allowing us to test whether value modulated neural signatures of semantic facilitation (N400), prediction violation (AP), or message-level processing (FN).

### 2.1 Method

#### 2.1.1 Participants

Power calculations were based on effect sizes from Lai et al. (2024), which used the same sentence materials to characterize the N400, AP, and FN effects. Using the smallest relevant effect size from that study, we estimated that 48 participants would provide at least 80% power to detect the expected ERP effects at α = 0.05. Accordingly, data are reported from 48 participants who were recruited from the Urbana-Champaign community. Participants were between 18 and 28 years old (Mean = 20.60, SD= 2.46, range = 18–28, 34 self-reported as female) and right-handed native English speakers with normal or corrected-to-normal vision and no history of neurological disorder. Participants were given course credit or monetary compensation for their participation. One additional participant was recruited but dropped prior to analysis due to a self-report that she did not understand the value manipulation.

#### 2.1.2 Stimuli and design

Participants studied value-cued sentences and were later tested with both free recall of the sentences and yes/no recognition of individual words from the study phase, including sentence-final words, sentence-medial words, new words, and lure words.

The sentence stimuli used in this study were identical to those in Chung and Federmeier (2023); key points are reproduced here and more detail can be found in that paper. 96 sentence frames were selected from a larger set of normed materials developed by Federmeier et al. (2007). In the norming study, native English speakers completed each sentence fragment with the word they would generally expect and with two additional possible completions, allowing cloze probabilities to be estimated not only for the best completion but also for plausible alternatives. Strongly constraining frames were those for which the best completion had high cloze probability, whereas weakly constraining frames had lower-cloze best completions; unexpected endings were selected to have near-zero cloze probability while remaining plausible in context. Critically, each unexpected ending was paired with both a strongly constraining and a weakly constraining frame, allowing responses to the same lexical item to be compared as a function of the strength of the prediction generated by the sentence context. Expected and unexpected endings were also chosen so that unexpected words came from a different semantic category than the expected ending and were minimally associated with either the expected ending or words in the sentence frame. Thus, the materials were constructed to isolate effects of message-level constraint and prediction violation, rather than effects driven by lexical properties of the critical final words, word association, or semantic feature overlap.

Sentences averaged 9.8 words in length (range: 4–19). Based on the (previously collected) cloze probability norms, each sentence frame was completed with both its most expected ending and with an unexpected but plausible completion with near-zero cloze probability (maximum cloze = 0.11). Half of the sentences were strongly constraining (cloze ≥0.68 for the expected completion); e.g., “His boss refused to give him a … raise (*Strong Constraint Expected; SCE*) / break (*Strong Constraint Unexpected; SCU*).” The other half were weakly constraining (cloze ≤ 0.42 for the expected completion); e.g., “Seth was uneasy because he had never liked to … lie (*Weak Constraint Expected; WCE)* / ski (*Weak Constraint Unexpected; WCU*).” Expected and unexpected sentence-final words were comparable in length, concreteness, imageability, familiarity, and frequency.

Stimuli were first divided into two lists, such that, within a list, each sentence frame appeared only once, and, across lists, each sentence appeared with both its expected and unexpected ending. Within each list, half of the frames for each constraint condition were completed by the expected ending and half were completed by the unexpected ending (yoked so that the matched unexpected endings did not appear in the same list). The value manipulation was adapted from value-directed remembering paradigms (e.g., Castel et al., 2002), although it was simplified relative to studies that use a broader range of point values. Specifically, we used two values, three and 10, to create low- and high-value sentence conditions while minimizing additional task demands and variability associated with using a larger value scale. Each sentence was thus associated with either a High value (10 points) or a Low value (3 points). Half of each constraint by expectancy condition (SCE, SCU, WCE, WCU) within each of the original two lists was assigned a High value and the other half a Low value, and then corresponding lists were created with the value assignment to each sentence swapped, yielding four final experimental lists. Stimulus characteristics were controlled for conditions across and within each list. Thus, participants saw each sentence frame and critical word only once, and, across lists, each sentence appeared with both its expected and unexpected completion and was paired with both value conditions.

For the recognition test, we used 192 single words: 48 sentence-final words, 48 words that had appeared in sentence-medial positions, 72 new words that had not appeared in any sentence, and 24 lure words corresponding to the expected completion of sentences that had instead ended unexpectedly. Across participants, the same word could serve as either a studied sentence-final item or a lure, depending on which sentence version had been presented. Lexical properties of the critical test items (Sentence Final, New, and Lure) were comparable across conditions. For each participant, half of the test items were old and half were new, with proportions of Sentence Medial (25%), Sentence Final (25%), New (37.5%), and Lure (12.5%) items held constant.

#### 2.1.3 Experimental procedure

Participants were seated in an electrically shielded EEG recording booth approximately 100 cm from a CRT monitor. The experimental procedure followed the Before condition of Chung and Federmeier (2023). Participants were instructed that their goal was to maximize their total point score by remembering as many sentences as possible. To encourage prioritization of valuable information, both written and verbal instructions emphasized that focusing on sentences associated with higher point values (10 points) would improve their overall score. Participants were also instructed to minimize movement and blinking during sentence presentation.

The experiment consisted of six study blocks, each containing 16 sentences (96 sentences total), counterbalanced across constraint, expectedness, and value conditions. After each study block, participants completed a free recall test. After every third recall test, participants also completed a recognition memory test. The experimental part of the session took approximately 1 h. To reduce fatigue and ensure attentiveness, participants were allowed to take breaks following recall and recognition tests.

##### 2.1.3.1 Study phase

Sentences were presented one word at a time using rapid serial visual presentation (RSVP). Each word appeared for 200 ms with a 300 ms interstimulus interval. This timing matches that in prior ERP studies using these same sentence materials with younger and older adults (Federmeier et al., 2007; Wlotko et al., 2012), thereby allowing the present N400, AP, and FN effects to be compared directly with prior findings. Each sentence was preceded by a fixation cross displayed for 200 ms. A numerical value indicating the sentence's importance (three points for low-value sentences, 10 points for high-value sentences) appeared for 3,000 ms prior to the fixation cross. The value cue was accompanied by the phrase “Next sentence:” and was displayed in color (orange for low value and blue for high value) to aid discrimination. A 3,000 ms interval separated successive sentences.

##### 2.1.3.2 Free recall

Following each study block, participants were asked to type the sentences they had just read in any order they could remember. Participants were encouraged to report complete sentences but were told that partial sentences or phrases were acceptable if they could not recall the entire sentence. Participants were allowed as much time as needed to complete the recall test before continuing to the next block.

##### 2.1.3.3 Recognition test

After every third block of sentences, participants completed a yes/no recognition test assessing memory for words in the preceding sentences. Test items were presented individually, and participants indicated whether they had seen each word in any of the sentences from the preceding blocks by selecting “Yes (seen word)” or “No (new word).” Each recognition test contained 96 items and participants were allowed unlimited time to respond.

#### 2.1.4 Coding and scoring

Coding and scoring of the recall data followed Chung and Federmeier (2023). Recall responses were coded offline by the first author, with ambiguous cases adjudicated with the second author. For descriptive purposes, responses were categorized based on their correspondence to the target sentence: *Verbatim* (exact reproduction of content words; e.g., *The police had never seen a man so drunk*.), *Almost verbatim* (minor surface changes but same message; e.g., *The cops had never seen someone so drunk*), *Gist* (accurate main message with reduced detail; e.g., *Police saw a man drunk*), *Fragment* (partial sentence lacking the main message; e.g., *Someone was very drunk*.), *Single word* (e.g., *police*), or *Unknown/Incorrect*. Because the primary interest was memory for the sentence's central meaning, analyses of recall data aggregated responses coded as Verbatim, Almost verbatim, and Gist, which all reflected accurate recall of the sentence's primary message.

During the experiment, participants received a value-conditionalized recognition score after each recognition block. Correct “yes” responses to previously presented words were awarded points corresponding to the value associated with the sentence in which the word had appeared. For offline analyses, recognition performance was calculated as the proportion of “Yes” responses to Sentence Final, Sentence Medial, New, and Lure items.

#### 2.1.5 EEG recording and processing

EEG data were recorded from 26 evenly spaced Ag/AgCl electrodes embedded in an EasyCap (Figure 1). Electrodes were also attached on each mastoid bone behind the ear, on the outer canthus of each eye (to monitor eye movements), and over the infraorbital ridge of each eye (to monitor blinks). All electrode impedances were kept under 5 kΩ. Signals were amplified using a BrainVision amplifier with a 16-bit A/D converter, an input impedance of 10 MΩ, a bandpass filter of 0.016–250 Hz, and a sampling rate of 1 kHz. The left mastoid electrode was used as a reference for online recording and data were re-referenced offline to the average of the left and right mastoids.

**Figure 1:**
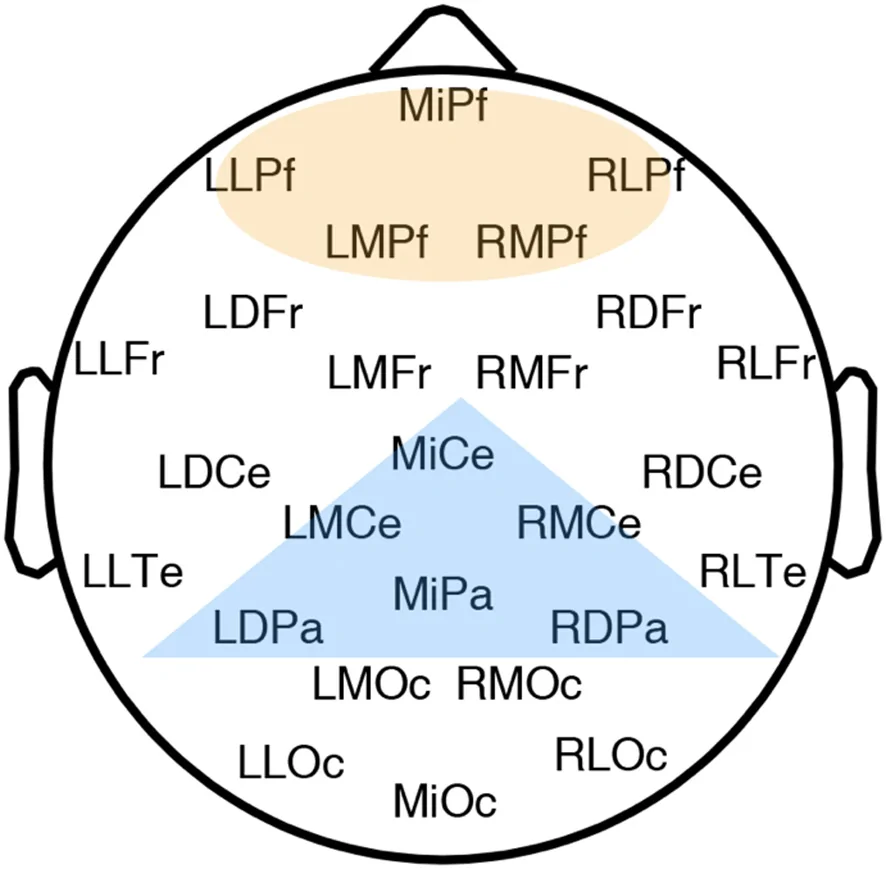
A schematic showing electrode positions with labels. The cluster of channels used for analysis are shaded. The top circle cluster of prefrontal channels was used to examine post-N400 frontal effects (AP and FN). The bottom triangle cluster of six centroparietal channels was used to examine the N400.

Following data collection and re-referencing, the continuous EEG was filtered offline through a 0.1–30 Hz Butterworth filter with a 12 dB/oct roll-off. ERPs were time-locked to the onset of the sentence-final word, which served as the critical word in the design. The signal was segmented into epochs from −200 to 1,000 ms post-stimulus-onset of each sentence-final word during encoding. The 200 ms pre-stimulus window was used for baseline correction of the post-stimulus data. Ocular artifacts were identified using a peak-to-peak function to detect blinks on a bipolar VEOG channel that was created by subtracting data in the lower left eye channel from the left prefrontal channel (LLPf). For subjects who had blinks for more than 10% of the trials, the data were run through AMICA (Palmer et al., 2012) and components with a high correlation between the time course of each component and the VEOG channel were removed from all trials. Remaining trials that contained blinks, eye movements, drift, or excessive muscle activity were removed with artifact thresholds calibrated by condition-blind visual inspection for each participant. On average, a total of 4.7% of the trials (SD = 3.4; range across participants: 1%−10.4%; no critical condition with more than 15% trial loss) were excluded from data analysis due to artifacts.

### 2.2 Event-related potentials and data analyses

ERPs to sentence-final words were measured by averaging at two time windows and two channel clusters in the time domain for each condition in each participant. Time windows were selected to be identical to those used in prior work with these materials (Federmeier et al., 2007; Wlotko et al., 2012), and an *a priori* region of interest was selected for each effect based on the distribution of that effect in those same studies. AP and FN amplitudes were measured in a 600–900 ms window, averaged across the five prefrontal channels (LLPf, MiPf, RLPf, LMPf, RMPf). Measurements of the mean N400 amplitude were collected from a 300–500 ms window across six centroparietal channels (LMCe, RMCe, MiCe, MiPa, LDPa, RDPa). Difference waves were constructed to extract critical effects of known direction. We examined the impact of expectancy on the N400 by subtracting the ERP waveforms of the unexpected ending from the expected ending separately for each level of constraint. The AP was examined as the difference between the SCU sentences (i.e., strongly constraining sentence frame with an unexpected completion) and the WCU sentences (i.e., weakly constraining sentence frame with an unexpected completion), creating a cloze-probability matched comparison of unexpected endings as a function of constraint. The FN was examined using a difference wave of the SCE sentences (i.e., strongly constraining sentence frame with an expected completion) and the same WCU control condition.

Statistical analyses on behavioral data were carried out by entering trial-level behavioral responses from recognition data (1 = judged “Seen” and 0 = judged “Not seen”) and recall data (1 = recalled and 0 = not recalled) into separate mixed effects logistic regression models in R version 4.0.3 (R Core Team, 2021), using the *glmer* function in the *lme4* package (Bates et al., 2015). Statistical analyses on ERPs were conducted by submitting participant-level difference waves to linear mixed effects regression models (using the *lmer* function), unless there was no interaction to be tested, which was the case for the late frontal effects averaged across all trials. For each late frontal effect, we first conducted a one-sample *t*-test of the average difference wave against zero (i.e., lack of effect) and then conducted an additional paired *t*-test to compare the average difference wave by value.

In the rare case where the linear mixed effects model with the simplest random structure (i.e., intercept-only model) did not converge, it was attributed to the lack of by-subject variance as evidenced by the random intercept for subjects being estimated at zero. Simple linear models with no random effects structures but the same fixed effects were then constructed instead using the *lm*() function (R Core Team, 2021), which did not change the model estimates. In all models, categorical predictors were contrast coded to allow factor coefficients to be interpreted as main effects. The random effects structure for all models reported was the maximal random effects structure that converged with random slopes that explained the least amount of variance being removed in cases of non-convergence (Barr et al., 2013). To ease convergence, correlations between random effects were not calculated. The specific fixed and random effects structures for each model are reported in the Results section below. Detailed statistical results for this experiment, including model estimates and confidence intervals, are provided in the Supplementary Material.

### 2.3 Results

#### 2.3.1 Behavioral data

##### 2.3.1.1 Recognition

*Overview of recognition responses*. Figure 2 shows the proportion of “Yes” responses for the four item types included in the recognition test. Sentence Medial and Sentence Final items were studied words, for which “Yes” was a correct response; Sentence Final items were the critical words manipulated by constraint, expectedness, and value, whereas Sentence Medial items provided a comparison measure of recognition for studied but noncritical words. New and Lure items were unstudied words, for which “Yes” was an incorrect response; Lure items corresponded to expected completions of sentences that had instead been presented with unexpected endings. Participants successfully discriminated studied sentence-final words from new items based on the average *d*', computed using the proportion of “Yes” responses to the critical Sentence Final items as hits and the proportion of “Yes” responses to New items as false alarms (mean *d*' = 1.37, range = 0.60–2.58).

**Figure 2:**
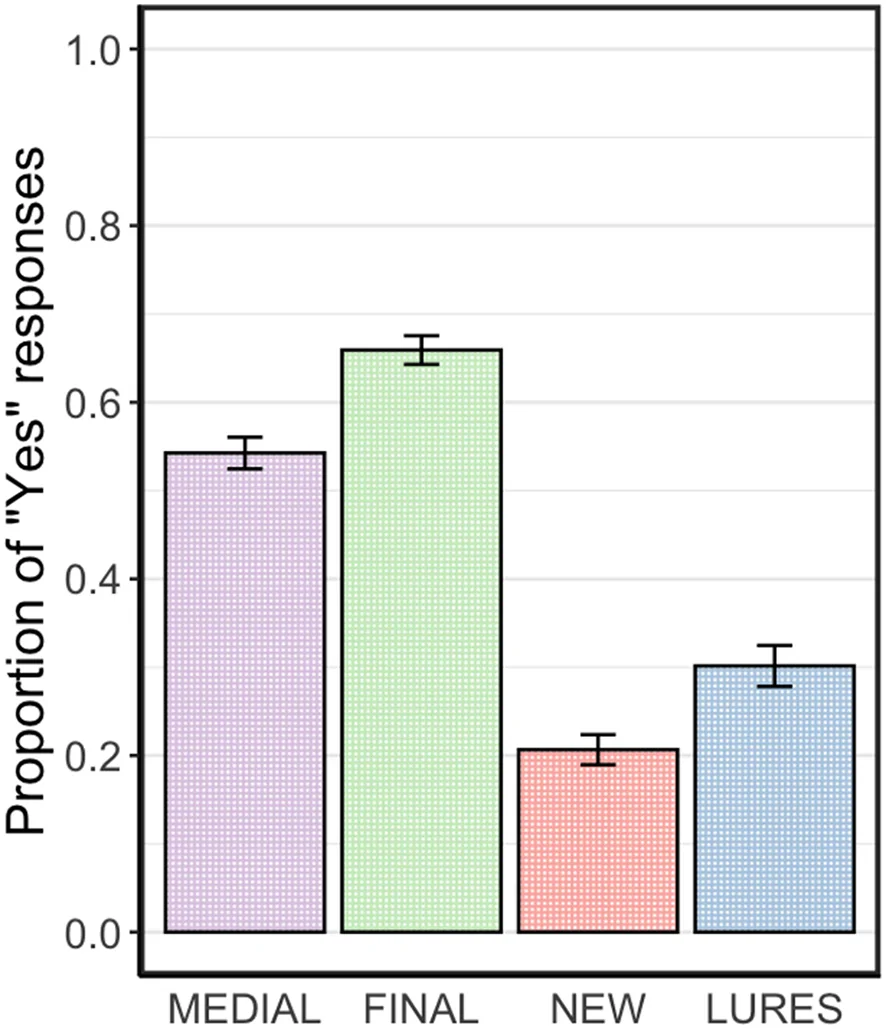
Overview of recognition responses in Experiment 1. Bars show the proportion of “Yes” responses for each recognition-test item type. Sentence Medial and Sentence Final items were studied words, for which “Yes” responses were correct; Sentence Final items were the critical manipulated words. New and Lure items were unstudied words, for which “Yes” responses were false alarms; Lure items were expected completions of sentences that had instead been presented with unexpected endings. Error bars reflect standard error around the mean.

*False alarms*. False alarms, defined as “Yes” responses to unstudied test items, were examined for New items, which had not appeared in any sentence, and Lure items, which were expected completions of sentences that had instead been presented with unexpected endings. Lures were further separated according to whether they came from strongly constraining sentence frames (SC Lures) or weakly constraining sentence frames (WC Lures). Trial-level behavioral responses were entered into a mixed effects logistic regression model with Value (High, Low), Category (New, SC Lure, WC Lure), and their interaction as fixed effects and random intercepts for participants and items but no by-participant or by-item slopes. Participants were more likely to falsely recognize Lure items than New items, β = −0.42, *z* = −3.18, *p* < 0.005, but false alarm rates did not differ reliably between SC and WC Lures, β = 0.22, *z* = 1.38, *p* = 0.17. False alarm rates did not differ as a function of Value, either overall or across item categories, all *p* > 0.55. Thus, as has been seen previously (Hubbard et al., 2019), participants showed false recognition for unseen items that were related to – and could have been predicted in – sentence frames that had been previously studied, without a reliable effect of sentential constraint.

*Hits*. We next examined hits to Sentence Final items, the studied critical words that had appeared as expected or unexpected endings of strongly or weakly constraining sentences. Trial-level responses for Sentence Final items were modeled with Value (High, Low), Constraint (Strong Constraint, Weak Constraint), Expectedness (Expected, Unexpected), and their interactions as fixed effects, with random intercepts for participants and items. In contrast with patterns observed in Chung and Federmeier (2023), participants recognized sentence-final words from high-value sentences (Mean =0.74, SD = 0.11) more often than those from low-value sentences (Mean =0.59, SD = 0.16), β = 0.73, *z* = 7.61, *p* < 0.001. However, participants were above chance at recognizing sentence-final words from low-value sentences, *t*(47) = 3.99, *p* < 0.001, indicating that they were not simply ignoring these sentences. The model also revealed a significant two-way interaction between Constraint and Expectedness, β = −0.86, *z* = −2.96, *p* < 0.005. Similar to the pattern seen in Chung and Federmeier (2023), Figure 3 shows that confirmed predictions (i.e., SCE endings) led to a reduced recognition rate compared to prediction violations (i.e., SCU endings) or sentence endings for weakly constraining sentences. The three-way interaction between Value, Constraint and Expectedness did not reach significance, β = −0.68, *z* = −1.77, *p* = 0.08 (the numerical pattern suggested that participants showed a tendency toward enhanced recognition for prediction violations in the high value condition), and no other main effects or interactions were significant, *p* > 0.12.

**Figure 3:**
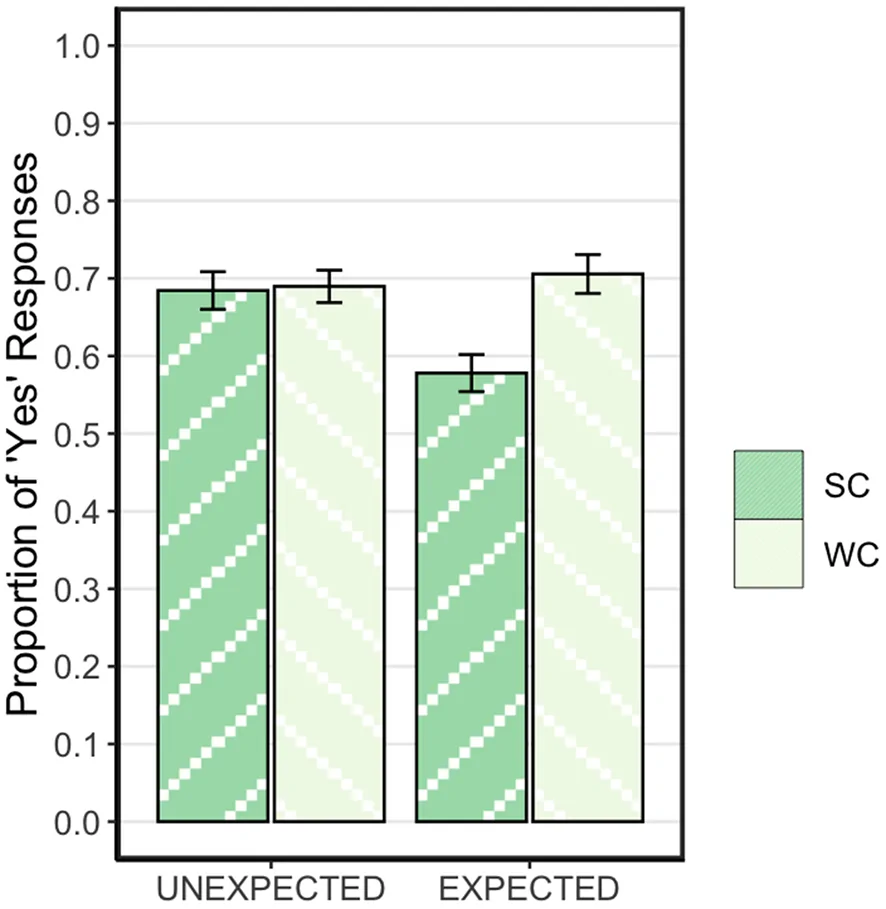
Recognition hits to sentence-final critical words in Experiment 1. Bars show the proportion of “Yes” responses to studied sentence-final words as a function of constraint and expectedness. SC, strongly constraining sentence frame; WC, weakly constraining sentence frame. Expected endings completed the sentence with the normatively expected word, whereas Unexpected endings were plausible but low-cloze completions. Error bars reflect standard error around the mean.

##### 2.3.1.2 Recall

*Overall performance*. Participants recalled an average of 28.31 sentences (SD = 9.75), corresponding to slightly less than one-third of the studied items.

*Impact of value*. To examine the impact of value on sentence memory, we constructed a logistic mixed-effects regression model that included fixed effects for Value (High, Low), Expectedness (Expected, Unexpected), and their interaction. The random effects structure included a random intercept for participants and items, random by-participant slopes for the main effect of value and random by-item slopes for the fixed effects. Participants successfully prioritized high value sentences irrespective of whether or not the sentence ended as expected (Figure 4), as revealed by a main effect of Value, β = 1.09, *z* = 5.69, *p* < 0.001, but no effect of Expectedness and no interaction of the two, *p* > 0.10.

**Figure 4:**
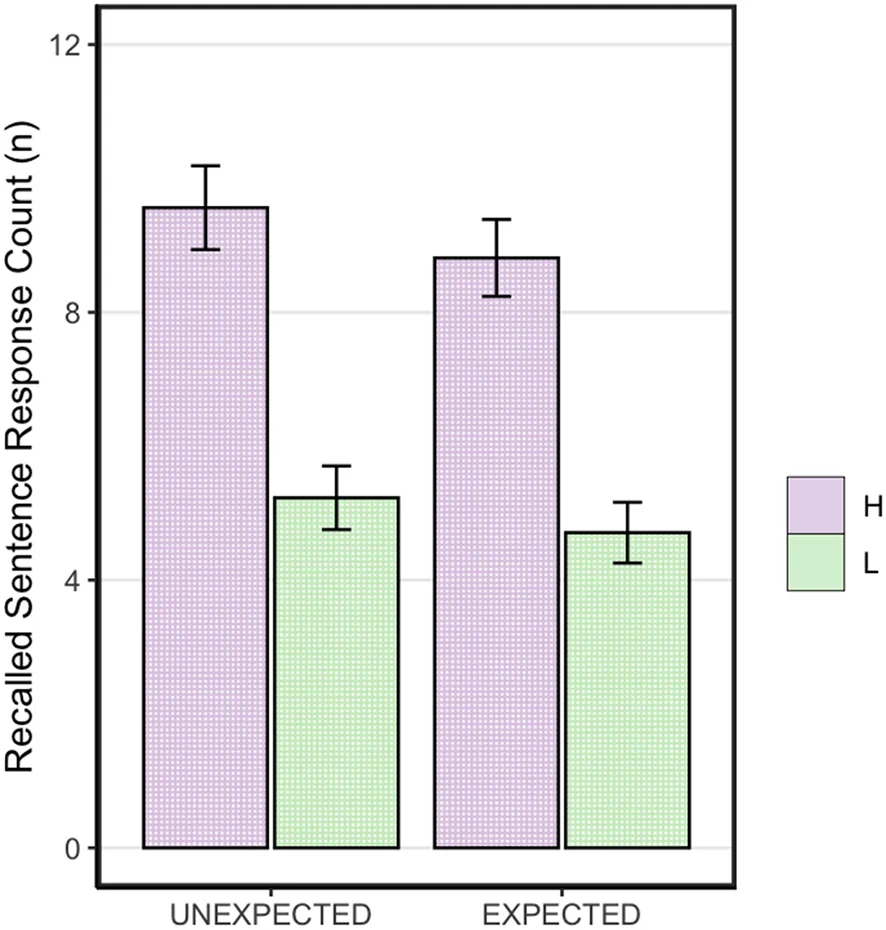
Sentence recall in Experiment 1. Bars show the number of sentences recalled as a function of value and expectedness of the sentence-final word. H = high value; L = low value. Unexpected sentences ended with plausible but low-cloze completions, whereas Expected sentences ended with the normatively expected completion. Error bars reflect standard error around the mean.

#### 2.3.2 ERPs

*All trials*. Figures 5, 6 show the grand average ERPs to the four types of sentence ending words (expected and unexpected endings of strongly and weakly constraining sentences), elicited in low and high value conditions, respectively.

**Figure 5:**
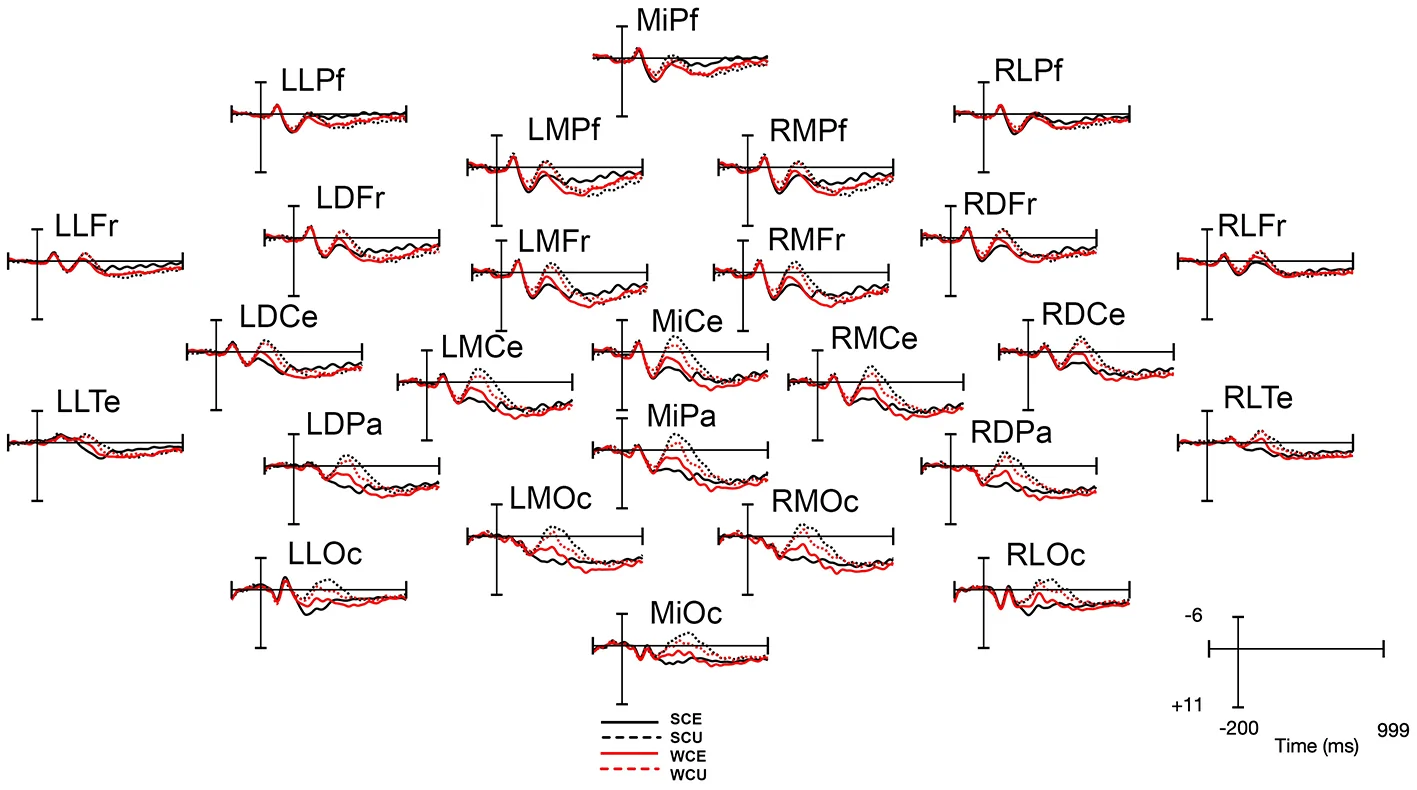
Grand-average ERPs to sentence-final critical words in low-value sentences in Experiment 1. ERPs are time-locked to the onset of the sentence-final word and shown at all scalp electrode sites. Sentence-final words were expected or unexpected completions of strongly constraining sentences (SCE and SCU, respectively) or expected or unexpected completions of weakly constraining sentences (WCE and WCU, respectively). Negative is plotted up.

**Figure 6:**
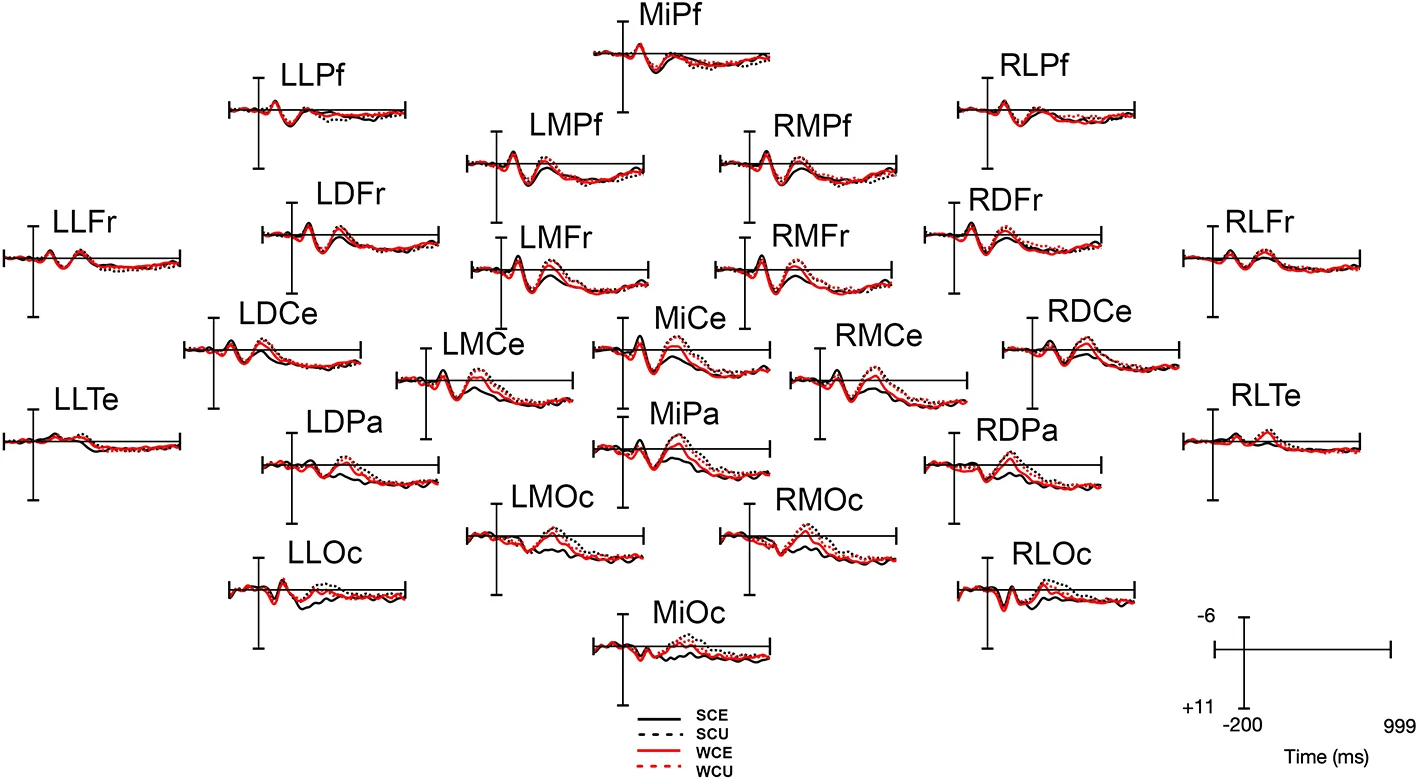
Grand-average ERPs to sentence-final critical words in high-value sentences in Experiment 1. ERPs are time-locked to the onset of the sentence-final word and shown at all scalp electrode sites. Sentence-final words were expected or unexpected completions of strongly constraining sentences (SCE and SCU, respectively) or expected or unexpected completions of weakly constraining sentences (WCE and WCU, respectively). Negative is plotted up.

##### 2.3.2.1 Anterior positivity

The AP was examined by comparing unexpected endings in strongly constraining sentences with unexpected endings in weakly constraining sentences (SCU–WCU), thereby isolating the effect of violating a strong contextual prediction while comparing endings that were unexpected in both conditions. Unexpected endings in strongly constraining sentences elicited more positive responses over prefrontal electrode sites between 600 and 900 ms than unexpected endings in weakly constraining sentences, *t*(47) = 2.02, *p* < 0.05. However, the effect did not differ by value, *t*(47) = −0.25, *p* = 0.81; see Figure 7.

**Figure 7:**
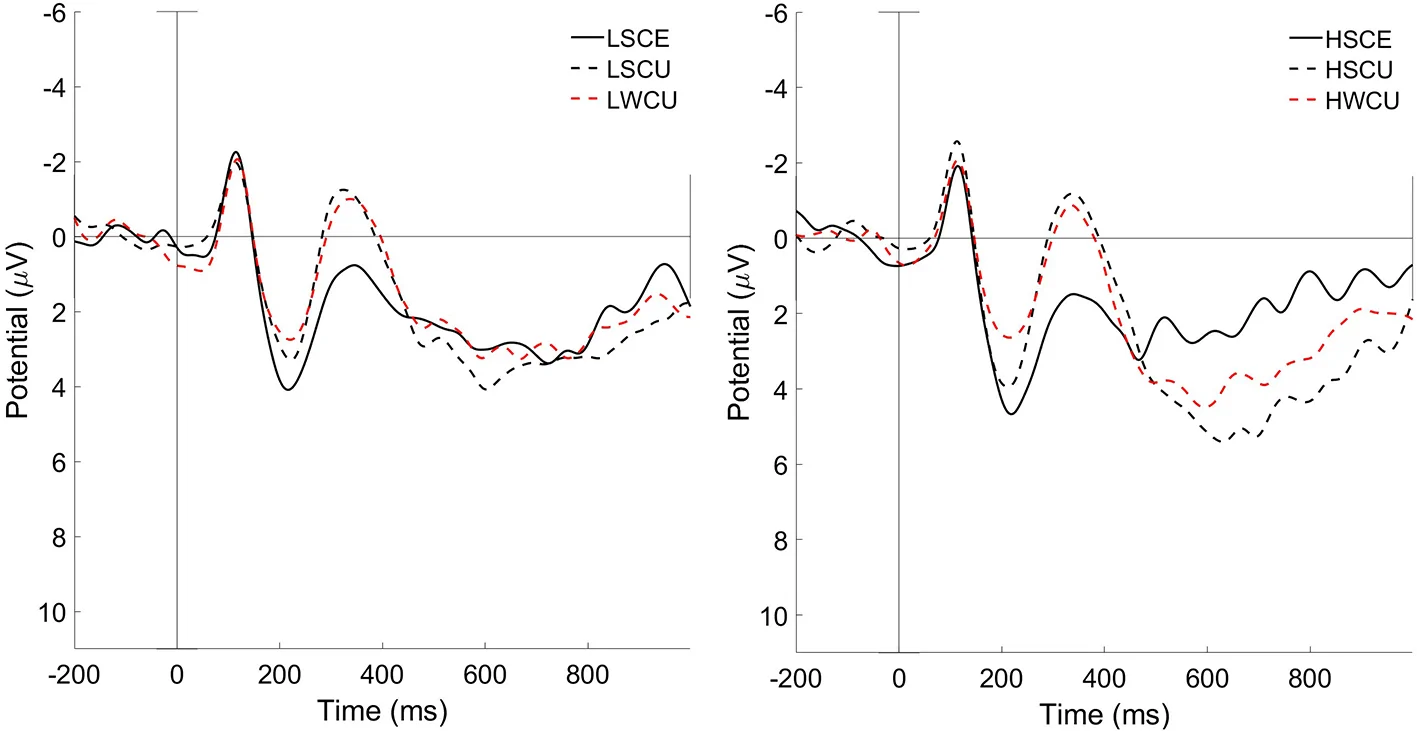
Late prefrontal ERP effects in Experiment 1. Waveforms from representative prefrontal channel LMPf show the contrasts used to isolate the anterior positivity (AP; SCU–WCU) and frontal negativity (FN; SCE–WCU) for low-value **(L)** sentences **(left)** and high-value **(H)** sentences **(right)**. Negative is plotted up. An AP effect is evident for both value conditions, with more positive responses to SCU endings. A larger FN effect (more negative response to SCE endings) can be observed for high value sentences.

##### 2.3.2.2 Frontal negativity

The FN was examined by comparing expected endings in strongly constraining sentences with unexpected endings in weakly constraining sentences (SCE–WCU), a contrast used to index late message-level processing associated with strongly expected completions. Across value conditions, SCE endings elicited numerically more negative responses over frontal electrode sites between 600 and 900 ms than did WCU endings, but the FN effect was not significantly different from zero overall, *t*(47) = −1.30, *p* = 0.20. Critically, the FN differed by value, *t*(47) = −2.43, *p* < 0.05, with high value items eliciting a larger FN than low value items (High: Mean = −1.34, SD = 3.65; Low: Mean = 0.44, SD = 3.22); see Figure 7.

##### 2.3.2.3 N400

The N400 expectancy effect was examined by subtracting responses to unexpected endings from responses to expected endings, separately for strongly and weakly constraining sentences, and entering these differences into a linear mixed effects model with Constraint (Strong Constraint, Weak Constraint), Value (High, Low), and their interaction as fixed effects and random intercepts for participants and items. Participants showed greater N400 facilitation for strongly than weakly constraining contexts, β = 2.34, *t* = 3.96, *p* < 0.001, consistent with prior work using these materials. Importantly, participants also showed greater N400 facilitation for high-value than low-value items, β = 1.58, *t* = 2.67, *p* < 0.05, indicating that value enhanced contextual facilitation across constraint conditions. The Value × Constraint interaction was not significant, β = 1.00, *t* = 0.84, *p* = 0.40 (Figure 8).

**Figure 8:**
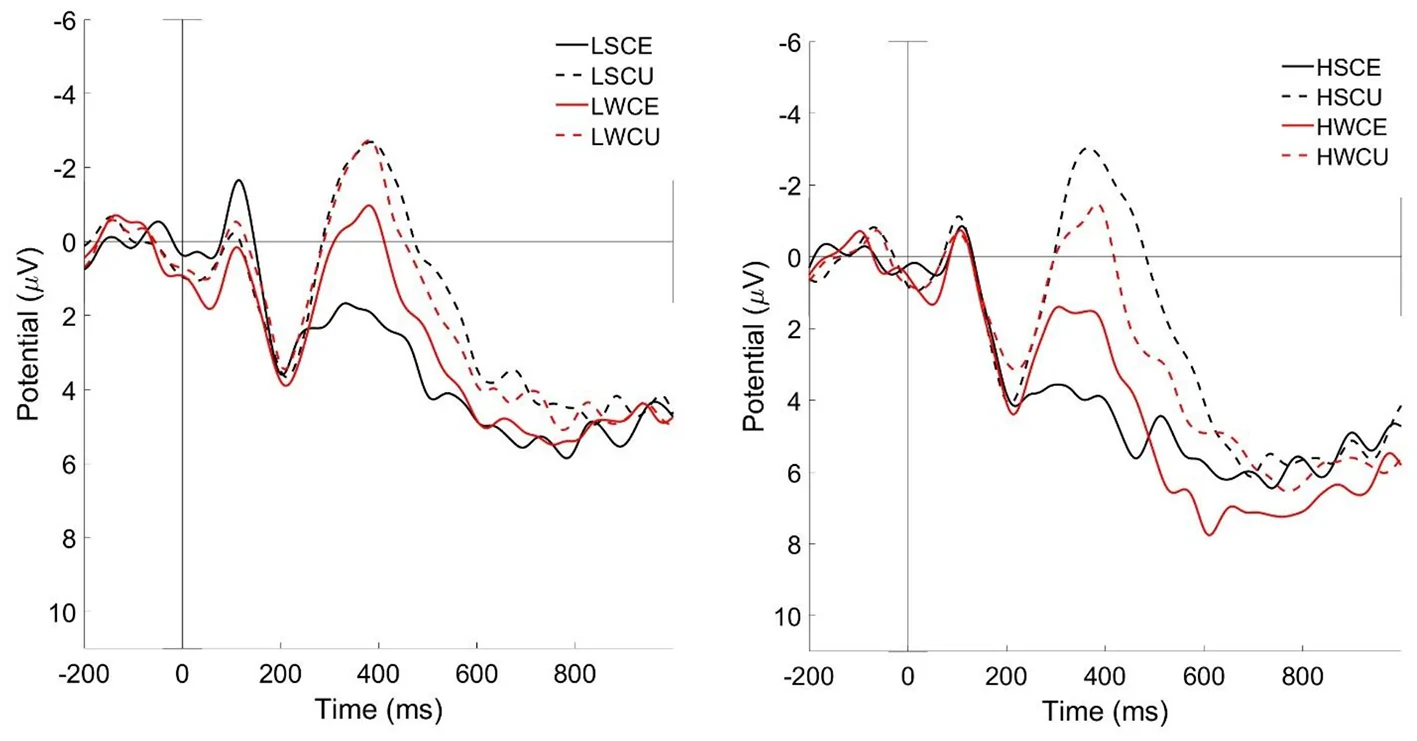
N400 expectancy effects in Experiment 1. Waveforms from representative centroparietal channel MiPa show responses to expected and unexpected sentence-final words in strongly and weakly constraining contexts, separately for low-value **(L)** sentences **(left)** and high-value **(H)** sentences **(right)**. SC = strong constraint; WC = weak constraint; E = expected; U = unexpected. Expected words elicited reduced N400s relative to unexpected words, with larger expectancy effects for strongly than weakly constraining sentences and enhanced expectancy effects for high-value sentences. Negative is plotted up.

##### 2.3.2.4 Trials backsorted by sentence-final word recall

To examine whether online processing at the sentence-final word was related to later memory, trials were backsorted into a “Recall” condition, containing trials in which participants subsequently recalled the sentence with its sentence-final word (or a synonym) and “No recall” trials in which participants did not provide any associated response during recall. Note that, different from the behavioral analyses, the recall bin here excluded trials for which participants recalled the main message of the sentence but not the final word (e.g., “Be sure to shuffle the cards first” after reading “Shuffle the cards before you forget”). This was to allow us to more directly link the impact of processing the sentence-final word to which the ERPs were time-locked to memory outcome.

Across all three ERP measures, there was no reliable relationship between neural activity at the sentence-final word and subsequent recall. For the AP (SCU–WCU), a linear mixed-effects model including Value and Recall Status revealed no main effects or interaction, all *p* > 0.31. Similarly, analyses of the FN (SCE–WCU) yielded no significant effects of Value, Recall Status, or their interaction, all *p* > 0.16. For the N400 expectancy effect (E–U), models conducted separately for strongly and weakly constraining sentences showed no effects of Recall Status or interactions with Value, all *p* > 0.11. A main effect of Value was observed for strongly constraining sentences, β = 2.80, *t* = 2.76, *p* < 0.01, reflecting enhanced facilitation for high-value items, but this effect did not interact with subsequent recall.

These analyses thus provide no evidence that variability in sentence-final word processing, as indexed by the AP, FN, or N400, predicted whether sentences were later recalled.

##### 2.3.2.5 Summary

Taken together, the ERP patterns indicate that younger adults routinely engaged predictive processing during sentence comprehension, as evidenced by the presence of an AP to unexpected endings in strongly constraining contexts that did not vary with value. At the same time, value cues modulated other aspects of processing: high-value sentences elicited greater semantic facilitation (larger N400 expectancy effects) and increased engagement of message-level processing (larger FN). These findings suggest that value influences the depth of processing during comprehension without necessarily altering the degree to which predictive mechanisms are engaged. Notably, however, variability in sentence-final ERP measures did not predict subsequent recall of the sentence message (including the sentence-final word), suggesting that the processes indexed at this position are not, on their own, sufficient to account for later memory outcomes.

### 2.4 Discussion

The goal of Experiment 1 was to use ERPs to examine how value cues influence processing during sentence comprehension. Replicating prior work using these materials (Chung and Federmeier, 2023), participants showed reliable sensitivity to value in their downstream memory: both recognition and recall were enhanced for high- relative to low-value sentences, indicating that participants successfully prioritized more important information during encoding. At the same time, recognition patterns bore the expected signatures of prediction-related processing (Chung and Federmeier, 2023; Hubbard et al., 2019; Hubbard and Federmeier, 2024). Participants were less likely to recognize confirmed predictions (strong constraint expected endings) than other sentence-final words, consistent with prior findings that predicted information is less likely to be encoded in a form that supports later recognition. In addition, participants showed elevated false recognition of lure items relative to new words, indicating sensitivity to predicted but unseen completions. Together, these behavioral patterns suggest that predictive processes were engaged during comprehension.

ERP measures provided converging evidence about the nature of these processes and how they were modulated by value. An AP was observed for unexpected endings in strongly constraining contexts, replicating a well-established signature of prediction violation (Federmeier et al., 2007; Otten and Van Berkum, 2008; Wlotko et al., 2012; Payne and Federmeier, 2017a). The size of this effect did not differ as a function of value, suggesting that the engagement of predictive processing itself was not strongly modulated by the importance of the sentence. This pattern is consistent with recent work suggesting that prediction-related processes indexed by the AP may not be graded by attention or task engagement (cf. Hubbard and Federmeier, 2021; Lai et al., 2024). In this sense, prediction appears to have been engaged broadly across conditions rather than selectively “ramped up” for high-value items.

In contrast, other aspects of processing were sensitive to value. High-value sentences elicited enhanced N400 facilitation, indicating greater use of contextual information to support semantic processing (Kutas and Federmeier, 2011; Federmeier, 2022). In addition, the FN was larger for high- than low-value items, consistent with increased engagement of message-level processing mechanisms (Wlotko and Federmeier, 2012b; Lai et al., 2024). Thus, although value cues did not appear to modulate whether predictive mechanisms were engaged, they did influence the depth or extent of semantic and integrative processing during comprehension.

Taken together, these findings suggest a dissociation between predictive processing and other forms of engagement during sentence comprehension. Predictive mechanisms, as indexed by the AP, were elicited regardless of value, whereas value selectively enhanced processes associated with semantic facilitation and message-level integrative processes. This pattern indicates that value cues do not simply increase overall processing effort, but instead shape specific components of comprehension in ways that may have distinct consequences for memory.

## 3 Experiment 2

The findings from Experiment 1 suggest that, for young adults, value cues do not modulate whether predictive mechanisms are engaged during comprehension, but instead influence other aspects of processing, including semantic facilitation and message-level integration. This dissociation raises an important question about how value-based prioritization operates in populations for whom the balance of these processes typically differs. In particular, older adults have been shown to be less likely to engage predictive processing mechanisms during sentence comprehension (Federmeier et al., 2010; Wlotko et al., 2012; Cheimariou et al., 2021) but often show preserved or even enhanced engagement of later integrative processes (Stine-Morrow et al., 2001, 2010; Wlotko and Federmeier, 2012a).

From this perspective, value cues may influence comprehension in older adults in ways that differ qualitatively from younger adults. If value primarily enhances processes such as semantic facilitation and message-level integration, older adults may be well positioned to benefit from value information. However, if value cues, particularly when provided in advance, can also prompt the engagement of predictive processing, then they may induce processing strategies that older adults do not typically employ. Critically, as suggested by prior work (Chung and Federmeier, 2023), such strategies may not always support accurate memory and may, under some conditions, even be detrimental.

Experiment 2 therefore examined how older adults use value information to guide memory for sentences and, in particular, whether the timing of value cues (before vs. after sentence presentation) differentially shapes memory outcomes. By manipulating when value information becomes available, this experiment tests whether older adults' memory for sentences and critical words in those sentences preferentially benefit from value cues that support later integrative processing, or whether advance value information leads them to adopt more active, prediction-based strategies similar to those observed in younger adults.

### 3.1 Method

#### 3.1.1 Participants

Participants were native English speakers (*M* = 66.05 years, SD = 6.09, age range: 55–81) who completed the experiment online. Participants were recruited from two sources. Half were drawn from a nationwide Prolific sample (www.prolific.co), matching the recruitment source used in Chung and Federmeier (2023), and half were recruited from the Urbana-Champaign community, providing a local older-adult sample demographically comparable to the participants recruited for the in-person ERP study in Experiment 3. We used both sources to facilitate comparison with the prior online young-adult behavioral study while also ensuring representation of the local population from which the ERP sample would be drawn. The Prolific sample also allowed us to avoid over-recruiting from the local older-adult pool before Experiment 3. Participants were recruited until a fully counterbalanced set of 48 was obtained from each sample. In both cases, participants completed the task online in one sitting in a quiet environment with a desktop or laptop computer and were paid $15 for attentive completion of the task. Participants recruited from the local community and Prolific did not differ in their recognition sensitivity as measured by their *d*'*, t*(88) = −0.61, *p* = 0.54, or their overall recall rate for sentences, *t*(94) = −1.42, *p* = 0.16 and so final analyses were conducted on the data collapsed across the two groups (total *N* = 96).

#### 3.1.2 Stimuli and experimental procedure

The experimental design and stimuli were identical to Chung and Federmeier (2023). We used the same behavioral procedures as in Experiment 1, with the exception that half of the participants always saw the value cue before the pre-trial fixation cross (Before condition) and the other half always saw the value cue after the last word of the sentence (After condition). The Before and After conditions were fully counterbalanced with the expectancy and value manipulations (i.e., an equal number of participants in each condition completed the four counterbalanced lists). Participants took 1 h on average to complete the task.

#### 3.1.3 Coding and scoring

Coding and scoring of the recall data were done using the same procedures as in Chung and Federmeier (2023); see description of the categories in Experiment 1. Recall responses were coded offline by the first author and an independent coder who was blind to the study hypotheses. Intercoder reliability was 97.38%. Responses that the two coders disagreed on were resolved through discussion between the first and second author.

### 3.2 Data analyses

Trial-level behavioral responses from recognition data (1 = judged “Seen” and 0 = judged “Not seen”) and recall data (1 = recalled and 0 = not recalled) from the Before and After conditions were each entered into separate mixed effects logistic regression models in R version 4.0.3 (R Core Team, 2021), using the *lme4* package (Bates et al., 2015). The categorical predictors were contrast coded to allow factor coefficients to be interpreted as main effects. The random effects structure for all models was always the maximal random effects structure that converged, with random slopes that explained the least amount of variance being removed in cases of non-convergence (Barr et al., 2013). The fixed and random effects for the specific models are reported in the Results section below. Detailed statistical results for this experiment, including model estimates and confidence intervals, are provided in the Supplementary Material.

### 3.3 Results

#### 3.3.1 Recognition

##### 3.3.1.1 Overview of recognition responses

Figure 9 plots the proportion of “Yes” responses for the four item types included in the recognition test, separately for the After condition, in which the value cue appeared after the sentence and the Before condition, in which the value cue appeared before the sentence and was therefore available during sentence encoding. Sentence Medial and (critical) Sentence Final items were studied words, for which “Yes” was a correct response; New and Lure items (expected completions of sentences that had instead been presented with unexpected endings) were unstudied words, for which “Yes” was an incorrect response. Overall discrimination, indexed by *d*', was similar in the Before condition (Mean = 1.72, SD = 0.74) as in the After condition (Mean = 1.79, SD = 0.53), *t*(85.83) = −0.50, *p* = 0.62.

**Figure 9:**
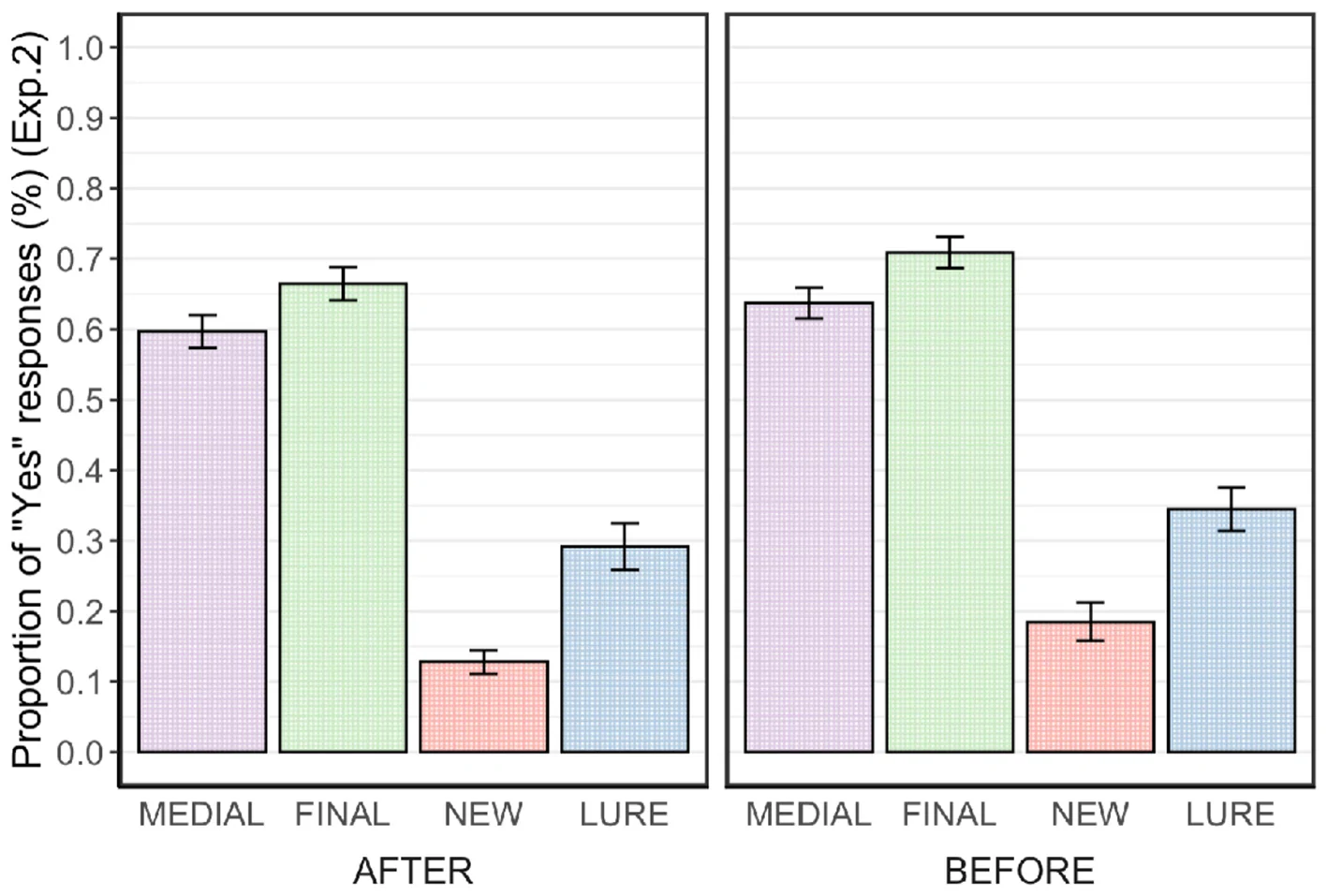
Overview of recognition responses in Experiment 2. Bars show the proportion of “Yes” responses for each recognition-test item type, separately for the After condition **(left)**, in which value appeared after the sentence, and the Before condition **(right)**, in which value appeared before the sentence. Sentence Medial and Sentence Final items were studied words, for which “Yes” responses were correct; Sentence Final items were the critical manipulated words. New and Lure items were unstudied words, for which “Yes” responses were false alarms; Lure items were expected completions of sentences that had instead been presented with unexpected endings. Error bars reflect standard error around the mean.

##### 3.3.1.2 False alarms

False alarms, defined as “Yes” responses to unstudied test items, included New items, which had not appeared in any sentence, and Lure items, which were expected completions of sentences that had instead been presented with unexpected endings. Lures were further separated according to whether they came from strongly constraining sentence frames (SC Lures) or weakly constraining sentence frames (WC Lures); Figure 10 plots the results for the After and Before conditions. Trial-level behavioral responses were entered into a mixed effects logistic regression model with Condition (After, Before), Category (New, SC Lure, WC Lure), and their interaction as fixed effects and random intercepts for participants and items. Participants were more likely to falsely recognize Lure items than New items, β = −0.52, *z* = −3.75, *p* < 0.001. False alarms did not differ reliably between SC and WC Lures, *p* = 0.08, and there was no significant interaction between condition and lure category, *p* = 0.06, although participants showed a numerical tendency to false alarm more frequently to SC Lures, which was slightly larger in the After condition. Thus, older adults in both conditions showed false recognition for lures that had been predictable in sentence frames that had been previously studied, with no reliable difference between SC and WC lures. Young adults in Chung and Federmeier (2023) did show a reliable constraint effect in the Before but not the After condition.

**Figure 10:**
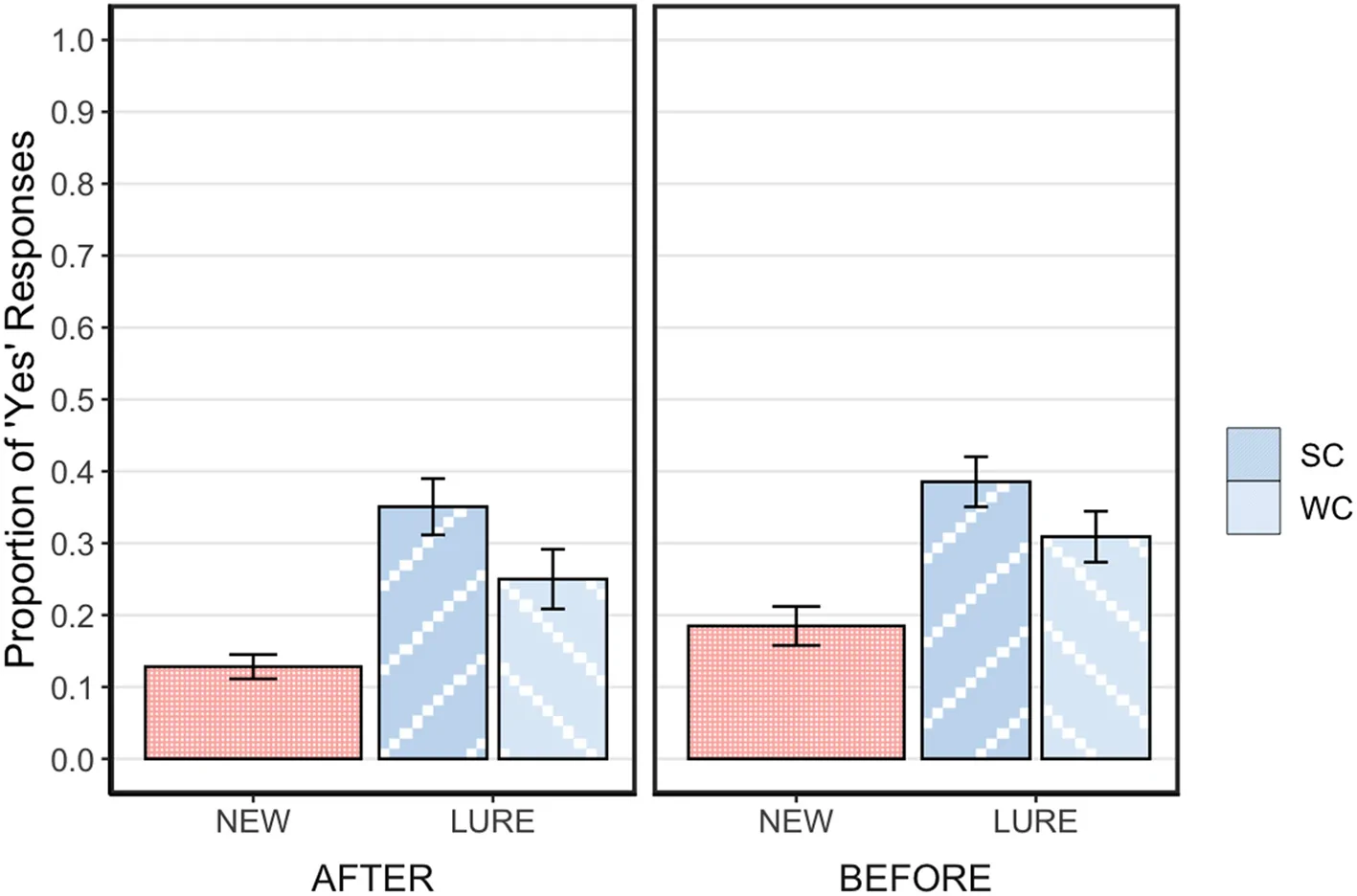
False alarms in Experiment 2. Bars show the proportion of “Yes” responses to unstudied recognition-test items, separately for the After condition **(left)** and Before condition **(right)**. New items had not appeared in any studied sentence. SC Lures and WC Lures were expected completions of strongly constraining and weakly constraining sentence frames, respectively, that had instead been studied with unexpected endings. SC = strong constraint; WC = weak constraint. Error bars reflect standard error around the mean.

##### 3.3.1.3 Hits

We first assessed whether value modulated recognition of Sentence Final items, the studied critical words that had appeared as expected or unexpected endings of strongly or weakly constraining sentences. In the After Condition, the hit rate for words from low-value sentences (Mean =0.65, SD = 0.18) did not significantly differ from those in high-value sentences (Mean =0.68, SD = 0.17), *t*(47) = 1.67, *p* = 0.10. Similarly, in the Before Condition, the hit rate for words from low-value sentences (Mean =0.69, SD = 0.19) did not significantly differ from its high-value counterpart (Mean =0.73, SD = 0.15), *t*(47) = 1.68, *p* = 0.10. This absence of a reliable value effect on recognition is consistent with the pattern observed for young adults in Chung and Federmeier (2023).

We next examined whether recognition of sentence-final critical words varied as a function of cue timing, constraint, and expectedness. Responses for Sentence Final items were entered into a mixed-effects model with Condition (After, Before), Constraint (Strong Constraint, Weak Constraint), Expectedness (Expected, Unexpected), and their interactions as fixed effects and with random intercepts for participants and items. Recognition patterns differed across the After and Before conditions as a function of Constraint and Expectedness, as reflected in a significant three-way interaction, β = −0.61, *z* = −2.18, *p* < 0.05. None of the remaining main effects or interaction terms were significant, *p* > 0.17. A visual inspection of the three-way interaction (Figure 11) suggested that the relationship between Constraint and Expectedness differed across the two Conditions, so two additional models were constructed to separately examine the impact of Constraint and Expectedness in the After and Before Conditions.

**Figure 11:**
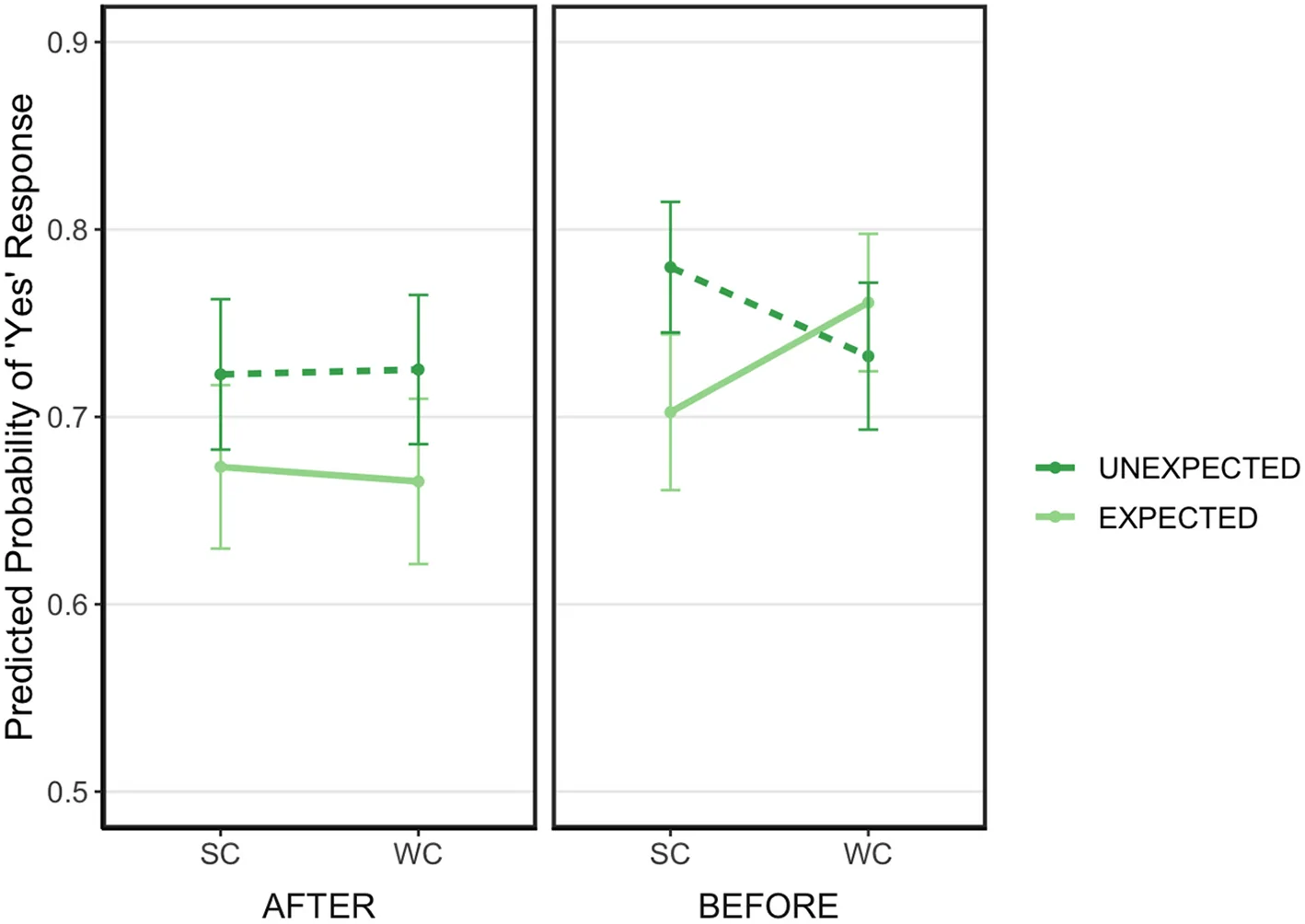
Recognition hits to sentence-final critical words in Experiment 2. Points indicate the proportion of correctly recognized sentence-final words, separately for the After condition **(left)** and Before condition **(right)**, as a function of constraint and expectedness. SC, strongly constraining sentence frame; WC, weakly constraining sentence frame. Expected endings completed the sentence with the normatively expected word, whereas Unexpected endings were plausible but low-cloze completions. Error bars reflect standard error around the mean.

The model assessing recognition of sentence final words in the After Condition included fixed effects for Constraint (Strong Constraint, Weak Constraint), Expectedness (Expected, Unexpected) and the interaction of the two, and converged with random intercepts for both items and participants. Although there was numerically a small overall memory boost for unexpected items, the model revealed no significant effect of Expectedness, Constraint, or an interaction of the two, *p* > 0.14.

In the Before condition, too, there was no main effect of Constraint or Expectedness, *p* > 0.37, and their interaction did not reach significance, β = −0.52, *z* = −1.69, *p* = 0.09. The numerical pattern suggested reduced recognition for prediction confirmations (i.e., expected words from strongly constrained sentence frames) relative to prediction violations (i.e., unexpected words from strongly constrained sentence frames), which was the pattern observed in Chung and Federmeier (2023).

#### 3.3.2 Recall

##### 3.3.2.1 Average recall rate across conditions

As an initial descriptive comparison of overall sentence recall across cue-timing conditions, we examined the number of studied sentences recalled in the After and Before conditions. Because we were interested in the impact of value on participants' ability to recall sentence-level information, analyses were limited to responses coded as verbatim, almost verbatim, or gist, as these contained accurate message-level information. Participants in the After Condition recalled approximately one quarter of the studied sentences (Mean = 25.42, SD = 11.97), similar to participants in the Before Condition (Mean = 27.71, SD = 15.1), and this difference was not significant, *t*(94) = −0.82, *p* = 0.42. Thus, knowing whether or not the upcoming sentence was important did not boost memory for sentences overall.

We then examined how value, cue timing, constraint, and expectedness shaped sentence recall at the trial level. Participants recalled more high-value than low-value sentences, indicating successful value-based prioritization in older adults. This was confirmed in a logistic mixed-effects regression model that included fixed effects for Condition (After, Before), Value (High, Low), Sentence Expectedness (Expected, Unexpected), and Constraint (Strong Constraint, Weak Constraint), and all interactions, with a random intercept for participants and items. The main effect of value was significant, β = 0.33, *z* = 6.43, *p* < 0.001, and no interactions containing Value were significant, *p* > 0.24, indicating that participants recalled more high-value than low-value sentences irrespective of Condition, Constraint, and Expectedness. The main effect of Expectedness did not reach significance, β = 0.09, *z* = 1.82, *p* = 0.07. However, recall was higher for strongly than weakly constraining sentences, β = 0.39, *z* = 3.09, *p* < 0.002, and this effect was qualified by a Constraint x Expectedness interaction, β = 0.49, *z* = 4.80, *p* < 0.001, reflecting a larger effect of constraint for sentences with expected completions than for sentences ending unexpectedly. No other main effects or interactions were significant, *p* > 0.23. Thus, across conditions, recall was enhanced for strongly constraining sentences that ended predictably (with a high cloze probability word), compared to strongly constraining sentence frames concluding with a prediction violation (Figure 12).

**Figure 12:**
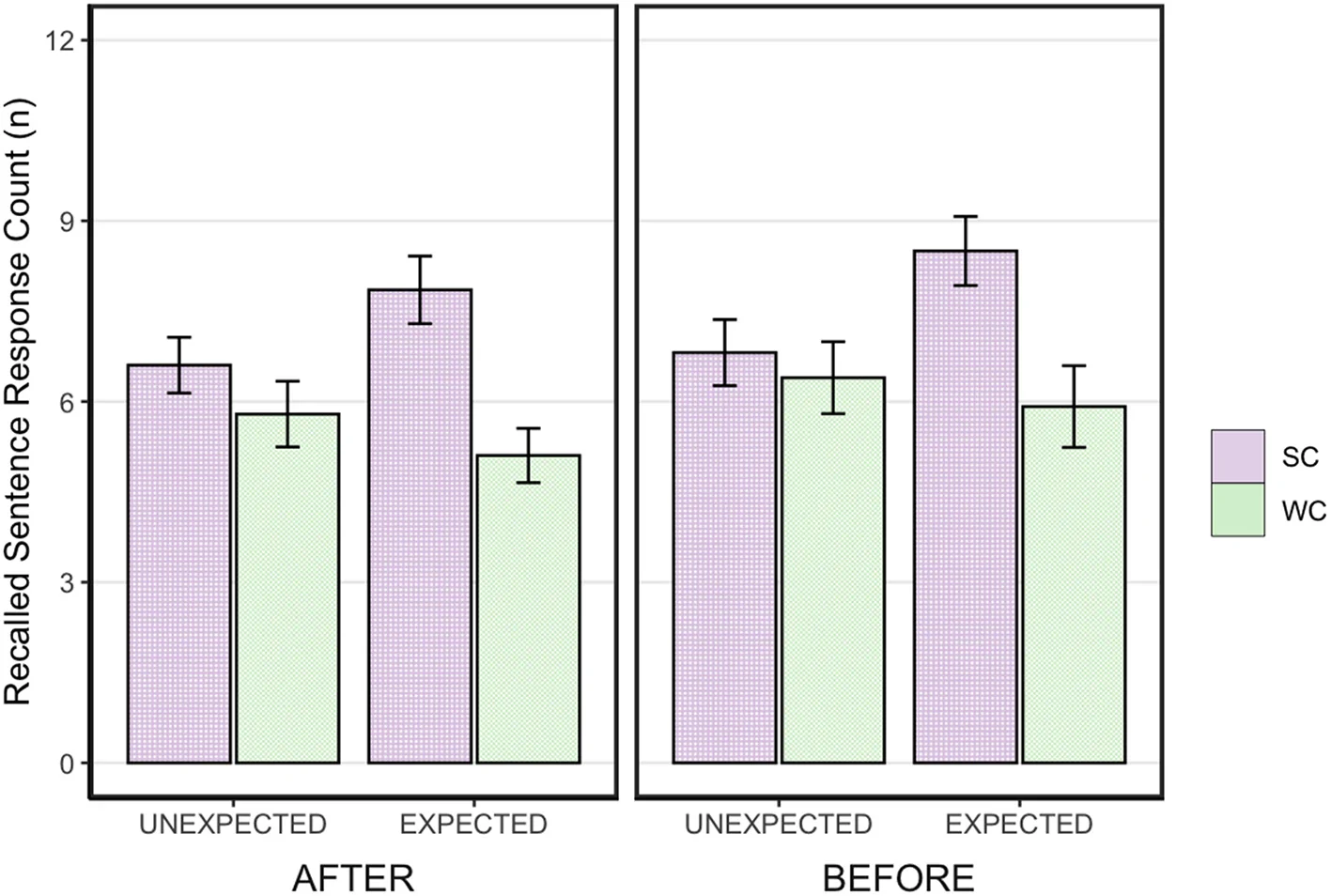
Sentence recall in Experiment 2. Bars show the number of recalled sentences in the After condition and Before condition as a function of cue timing, constraint, and expectedness of the sentence-final word. SC, strongly constraining sentence frame; WC, weakly constraining sentence frame. Expected sentences ended with the normatively expected completion, whereas Unexpected sentences ended with a plausible but low-cloze completion. Error bars reflect standard error around the mean.

### 3.4 Discussion

Experiment 2 examined whether older adults use value information to prioritize memory for sentences and whether the timing of value cues modulates this process. Overall, older adults showed reliable sensitivity to value, with better recall for high- than low-value sentences. This finding is consistent with prior work demonstrating preserved value-directed remembering in aging (e.g., Castel et al., 2002, 2013; Friedman et al., 2015) and extends it to more complex, sentence-level materials.

A central question was whether older adults would show the same pattern of strategic modulation by cue timing observed in younger adults by Chung and Federmeier (2023). In that study, memory outcomes differed across the Before and After conditions, suggesting that advance knowledge of value encouraged the use of different encoding strategies, potentially including those related to prediction. In contrast, older adults in the present study showed relatively similar patterns of memory performance across the two conditions. Recognition sensitivity did not differ as a function of cue timing, and overall recall rates were comparable for the Before and After conditions. Thus, unlike younger adults, older adults did not show strong evidence of qualitatively different memory outcomes as a function of when value information was provided.

At the same time, some aspects of performance indicate sensitivity to the predictive structure of the sentences. Older adults, like younger adults in Chung and Federmeier (2023), showed elevated false recognition of lure items relative to new items, suggesting that they were influenced by predictable but unseen completions. In addition, recall was better for strongly constraining sentences that ended with expected completions than for those that ended unexpectedly, indicating that coherent, predictable message-level representations support memory. However, these effects were not reliably modulated by cue timing, and evidence for condition-specific differences in recognition patterns was limited, as the overall three-way interaction in recognition was significant, but follow-up analyses within the separate cue-timing conditions were not. Thus, although older adults were sensitive to predictability, there was little indication that they flexibly adjusted their encoding strategies based on when value information became available.

Taken together, these findings suggest that older adults can successfully use value cues to prioritize memory but may be less likely than younger adults to specifically adapt their sentence processing strategies in response to value information. At the same time, the presence of prediction-consistent memory patterns raises the possibility that older adults may engage such processes under some conditions, even if they do not do so flexibly or selectively.

Experiment 3 therefore examines this possibility more directly by recording ERPs during sentence comprehension by older adults when value information was available in advance. This approach allows us to assess whether older adults engage prediction-related processes online, as indexed by the AP, and whether value cues modulate other aspects of comprehension, including semantic facilitation (N400) and message-level processing (FN), as observed in younger adults in Experiment 1.

## 4 Experiment 3

Experiment 3 was designed to directly assess the processing mechanisms that older adults engage when value information is available in advance of sentence comprehension. Older adults typically do not elicit prediction-related AP effects during passive comprehension, but it is possible that memory-based task demands and/or the availability of value information could lead older adults to engage predictive processing. If, in parallel to the pattern observed in younger adults, value affects processing in older adults by enhancing semantic facilitation and integrative processing, then we would expect to observe larger N400 expectancy effects and increased FN for high- relative to low-value sentences. Alternatively, as suggested by the behavioral patterns observed in Experiment 2, it is possible that older adults do not flexibly adjust their processing strategies as a function of value.

### 4.1 Method

#### 4.1.1 Participants

A total of 48 participants were recruited from the Urbana–Champaign community, matching the sample size of Experiment 1. Participants were between 55 and 77 years of age (*M* = 67.07, SD = 4.13; 34 self-identified as female) and were right-handed native English speakers with normal or corrected-to-normal vision. Five participants were excluded from analysis based on health conditions or medication use (*n* = 2) or failure to complete the task (*n* = 3). Because of an extended pandemic-related lab shutdown, additional participants could not be recruited to replace those excluded, so the final sample size was 43.

#### 4.1.2 Stimuli and design

The stimuli and design were identical to Experiment 1.

#### 4.1.3 Experimental procedure

The experimental procedure was largely identical to Experiment 1, with minor adjustments to facilitate task performance in older adults. First, participants were shown a brief demonstration of the RSVP sentence presentation prior to the start of the experiment and were allowed to adjust their viewing distance to ensure comfortable reading. Second, although participants were encouraged to minimize blinking and movement during sentence presentation, they were instructed to blink as needed if doing so improved task comfort and focus.

#### 4.1.4 Coding and scoring

Coding and scoring of behavioral data were identical to Experiment 1.

#### 4.1.5 EEG recording and processing

EEG recording and processing were identical to Experiment 1. On average, a total of 3.1% of the trials (SD = 2.4; range across participants: 1%−9.4%; no critical condition exceeded 13% trial loss) were excluded from data analysis due to artifacts.

### 4.2 Event-related potentials and data analyses

ERP quantification and statistical analyses were identical to those described in Experiment 1. Detailed statistical results for this experiment, including model estimates and confidence intervals, are provided in the Supplementary Material.

### 4.3 Results

#### 4.3.1 Behavioral data

##### 4.3.1.1 Recognition

*Overview of recognition responses*. Figure 13 provides an overview of recognition performance for the older adults in Experiment 3. As in Experiment 1, the test included studied words from sentence-medial and sentence-final positions, along with unstudied New words and Lure words. “Yes” responses were correct for the studied items and false alarms for the unstudied items. Sentence Final items were the critical words that varied in constraint, expectedness, and value, and Lure items were expected completions of sentences that had actually been read with unexpected endings. Participants successfully discriminated between studied sentence-final words and new items (mean *d*' = 1.44, range: 0.80–2.83).

**Figure 13:**
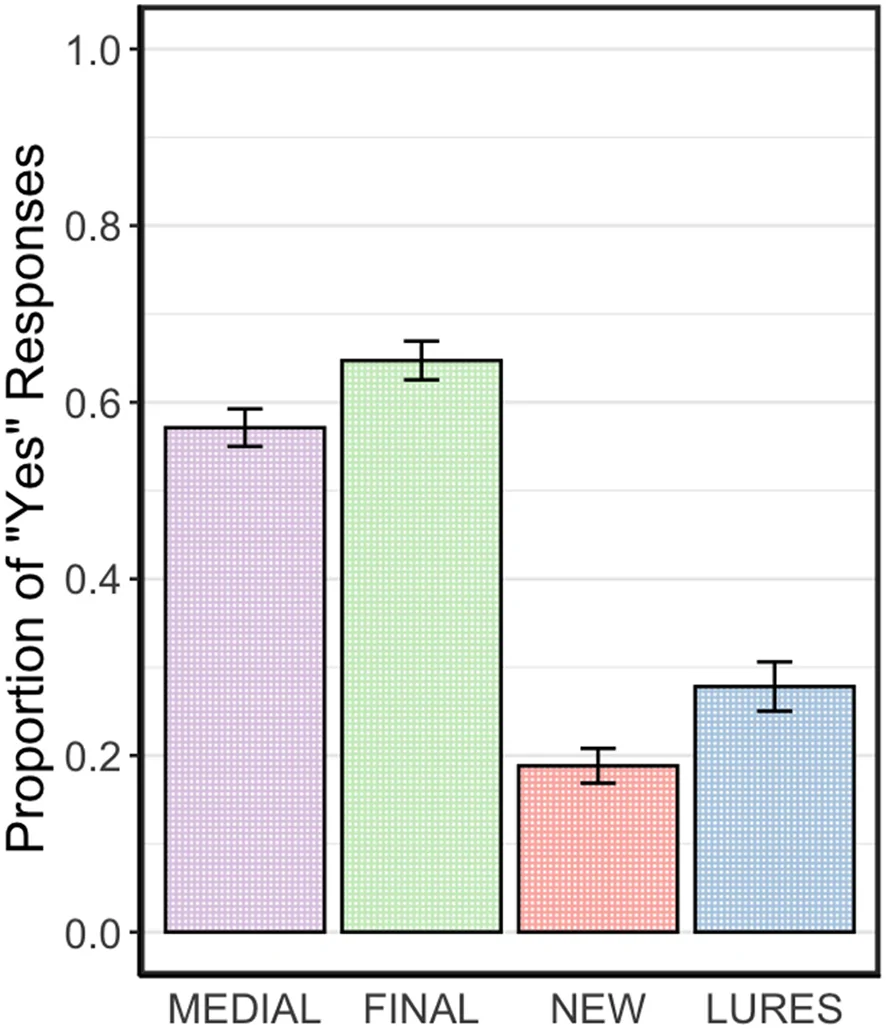
Overview of recognition responses in Experiment 3. Bars show the proportion of “Yes” responses for each recognition-test item type. Sentence Medial and Sentence Final items were studied words, for which “Yes” responses were correct; Sentence Final items were the critical manipulated words. New and Lure items were unstudied words, for which “Yes” responses were false alarms; Lure items were expected completions of sentences that had instead been presented with unexpected endings. Error bars reflect standard error around the mean.

*False alarms*. We next considered older adults' false recognition of unstudied test words, including New items that had not appeared in any studied sentence, and Lure items, which were separated according to whether they came from strongly constraining sentence frames (SC Lures) or weakly constraining sentence frames (WC Lures). Trial-level responses were entered into a mixed-effects logistic regression model with Value (High, Low), Category (New, SC Lure, WC Lure), and their interaction as fixed effects, along with random intercepts for participants and items and a by-participant slope for Value. Older adults were more likely to falsely recognize Lure items than New items, β = −0.77, *z* = −3.42, *p* < 0.001, indicating sensitivity to predicted but unseen completions. They also false alarmed more often to SC than WC Lures, β = 1.09, *z* = 2.98, *p* < 0.004. This SC–WC difference was larger for high- than low-value items, as reflected in the Value × Category effect, β = −0.80, *z* = −2.14, *p* < 0.04 (Figure 14). No other effects were significant, *p* > 0.35. Thus, different from young adult patterns in Experiment 1, older adults showed a sensitivity to constraint in their false alarms to lures (mirroring the numerical pattern seen in Experiment 2).

**Figure 14:**
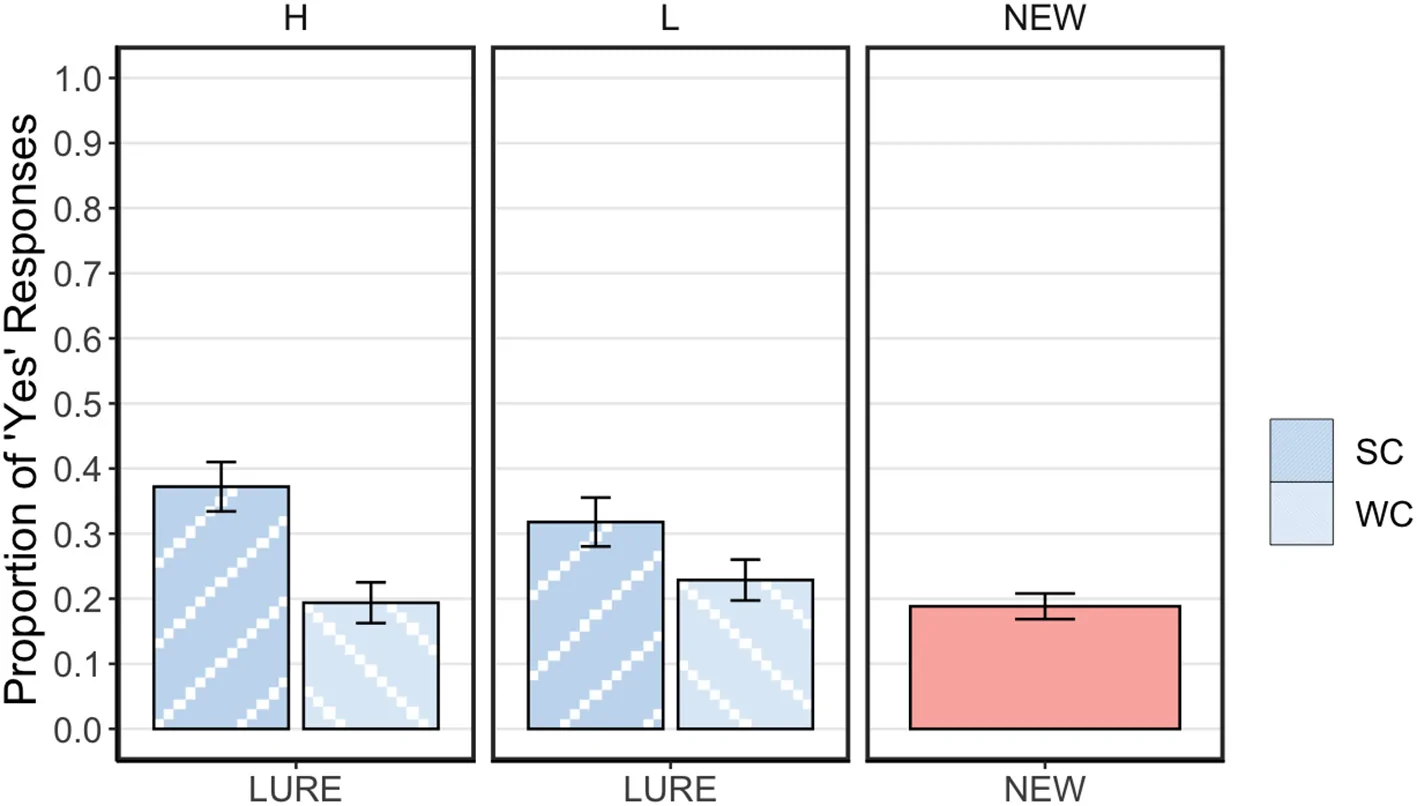
False alarms in Experiment 3. Bars show the proportion of “Yes” responses to unstudied recognition-test items as a function of value and item type. New items had not appeared in any studied sentence. SC Lures and WC Lures were expected completions of strongly constraining and weakly constraining sentence frames, respectively, that had instead been studied with unexpected endings. H = high value; L = low value; SC, strong constraint; WC, weak constraint. Error bars reflect standard error around the mean.

*Hits*. Recognition of Sentence Final items provided a measure of memory for the critical studied words, which had appeared as expected or unexpected completions of strongly or weakly constraining sentence frames. A mixed-effects model, including fixed effects of Value (High, Low), Constraint (Strong Constraint, Weak Constraint), Expectedness (Expected, Unexpected), and the resulting interactions and a random intercept for participants and items, revealed a main effect of Value, β = 0.52, *z* = 4.60, *p* < 0.001. In contrast to the pattern observed in the online behavioral studies (Chung and Federmeier, 2023; Experiment 2), but consistent with the in-lab findings for younger adults (Experiment 1), recognition was higher for high-value (Mean =0.69, SD = 0.05) than low-value items (Mean =0.60, SD = 0.07), *t*(42) = 3.68, *p* < 0.001. Participants were nevertheless above chance in recognizing low-value items, *t*(42) = 3.85, *p* < 0.001, indicating that low-value sentences were not ignored. Value did not interact with other factors, *p* > 0.23. There was also a main effect of Expectedness, β = −0.40, *z* = −2.29, *p* < 0.03, driven by reduced recognition for prediction-confirming endings (i.e., SCE items; Figure 15). No other effects were significant, *p* > 0.14. This pattern mirrors that observed in Experiment 1, in which confirmed predictions were less likely to be recognized, and suggests that prediction-related encoding biases were present in the current sample.

**Figure 15:**
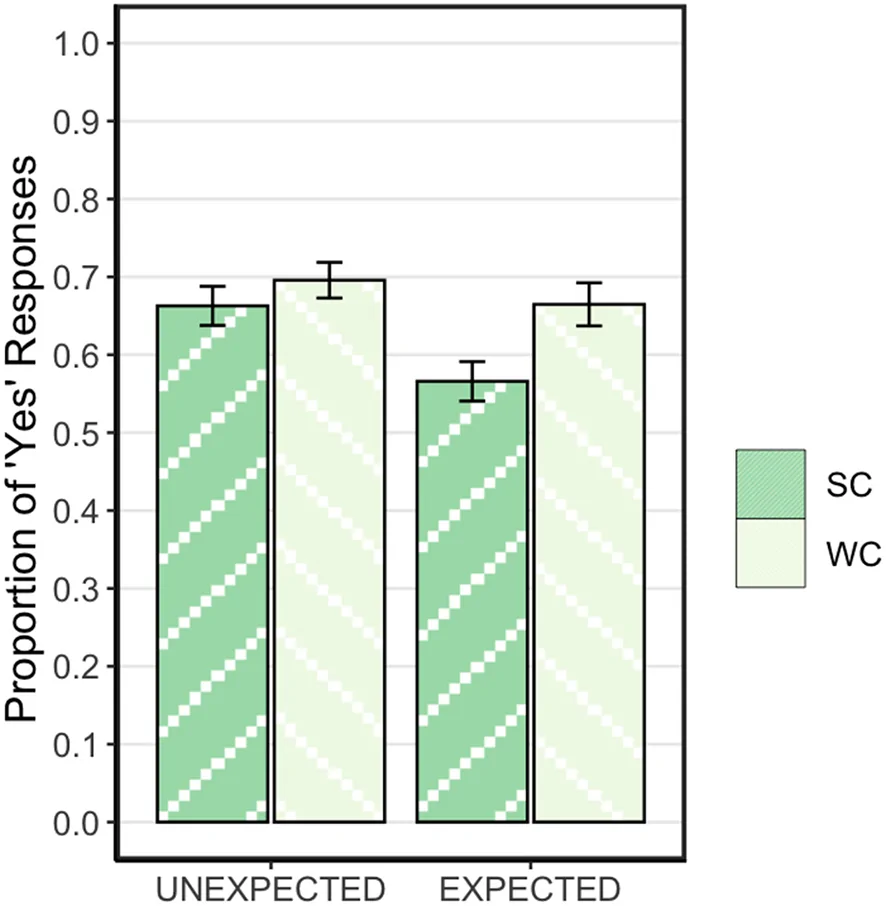
Recognition hits to sentence-final critical words in Experiment 3. Bars show the proportion of “Yes” responses to studied sentence-final words as a function of value, constraint, and expectedness. SC, strongly constraining sentence frame; WC, weakly constraining sentence frame. Expected endings completed the sentence with the normatively expected word, whereas Unexpected endings were plausible but low-cloze completions. Error bars reflect standard error around the mean.

##### 4.3.1.2 Recall

*Overall performance*. Participants recalled, on average, slightly less than one quarter of the studied sentences (Mean = 23.49, SD = 9.41).

*Impact of value*. Recall performance was analyzed using a mixed-effects logistic regression model with fixed effects of Value (High, Low), Constraint (Strong Constraint, Weak Constraint), Expectedness (Expected, Unexpected), and their interactions, along with random intercepts for participants and items and by-item slopes for Value and Expectedness. Older adults recalled high-value sentences more often than low-value sentences, β = 0.73, *z* = 6.58, *p* < 0.001, indicating successful value-based prioritization in sentence recall. Recall was also higher for strongly than weakly constraining sentences, β = 0.45, *z* = 3.02, *p* < 0.004. No other effects were significant, *p* > 0.24. Figure 16 shows the pattern. Thus, as in Experiment 2 – and paralleling effects in young adults in Experiment 1 – older adults successfully prioritized high-value information during recall of sentences. In addition, sentence-level constraint supported memory independently of value, with no evidence that value modulated the impact of expectedness or constraint on recall.

**Figure 16:**
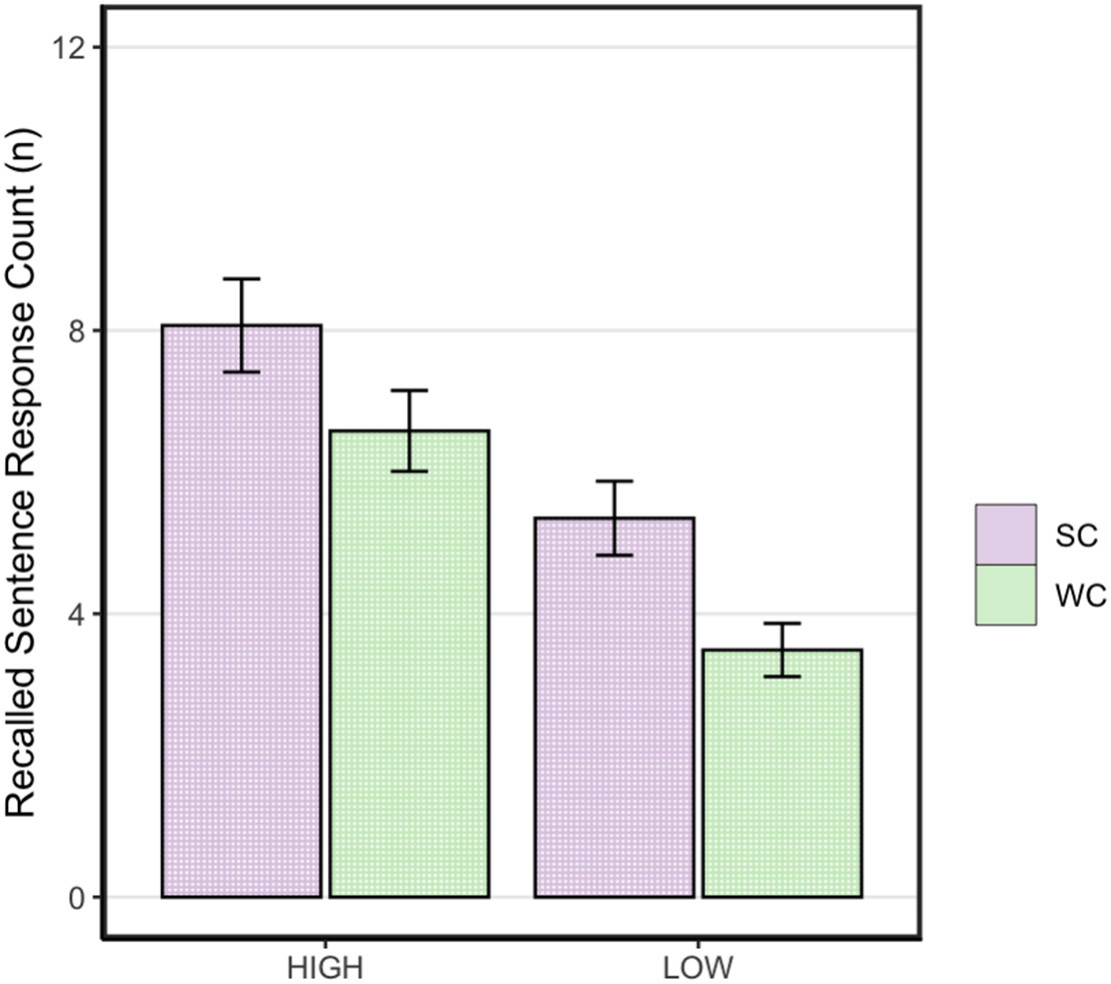
Sentence recall in Experiment 3. Bars show the number of recalled sentences as a function of value (high and low), and constraint of the sentence frame. SC = strongly constraining sentence frame; WC = weakly constraining sentence frame. Error bars reflect standard error around the mean.

#### 4.3.2 ERPs

##### 4.3.2.1 All trials

We next examined older adults' ERPs to the sentence-final critical words presented during encoding. These final words were expected or unexpected completions of strongly or weakly constraining sentence frames, allowing us to examine the N400, AP, and FN as a function of contextual constraint, expectedness, and value. Figures 17 and 18 show the grand average ERPs to the four types of sentence ending words, elicited in low and high value sentences respectively.

**Figure 17:**
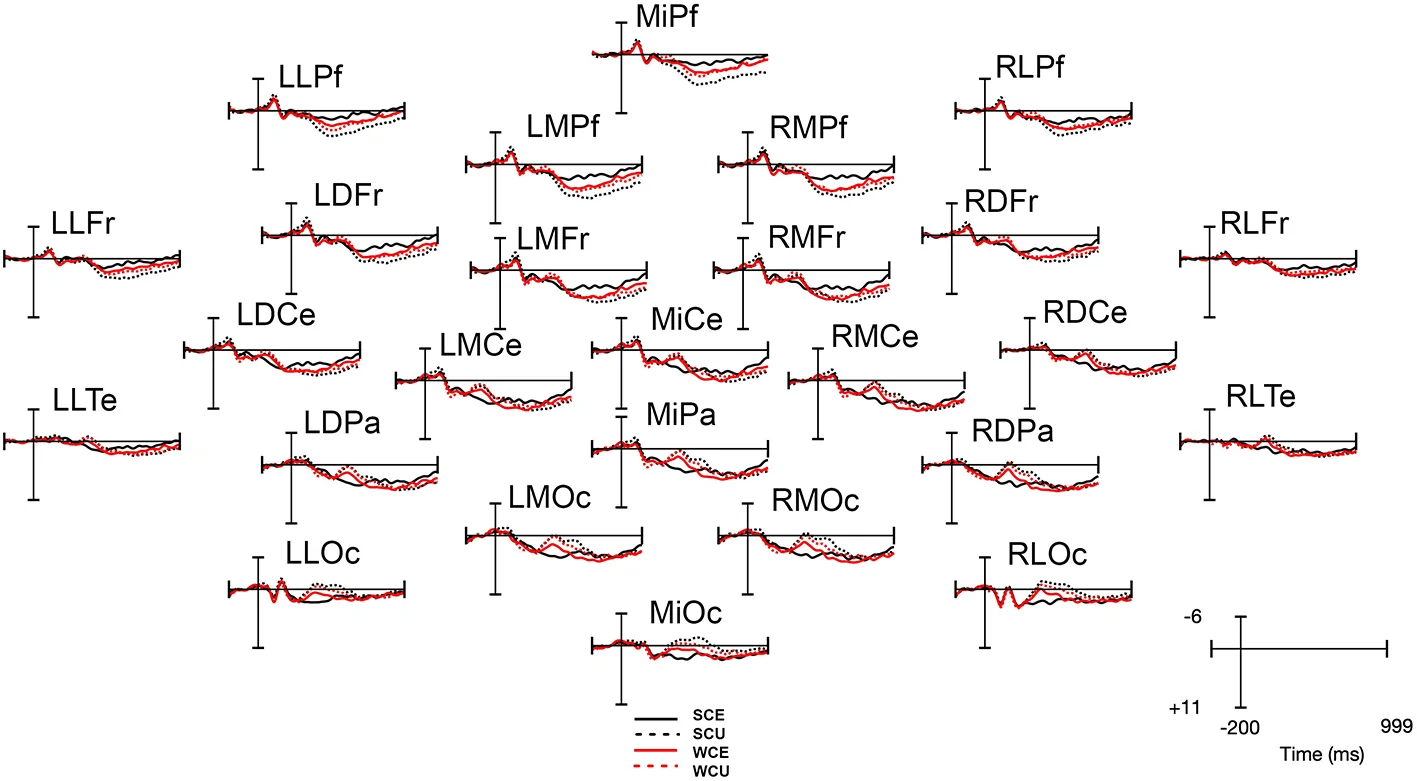
Grand-average ERPs to sentence-final critical words in low-value sentences in Experiment 3. ERPs are time-locked to the onset of the sentence-final word and shown at all scalp electrode sites. Sentence-final words were expected or unexpected completions of strongly constraining sentences (SCE and SCU, respectively) or expected or unexpected completions of weakly constraining sentences (WCE and WCU, respectively). SC, strong constraint; WC, weak constraint; E = expected; U = unexpected. Negative is plotted up.

**Figure 18:**
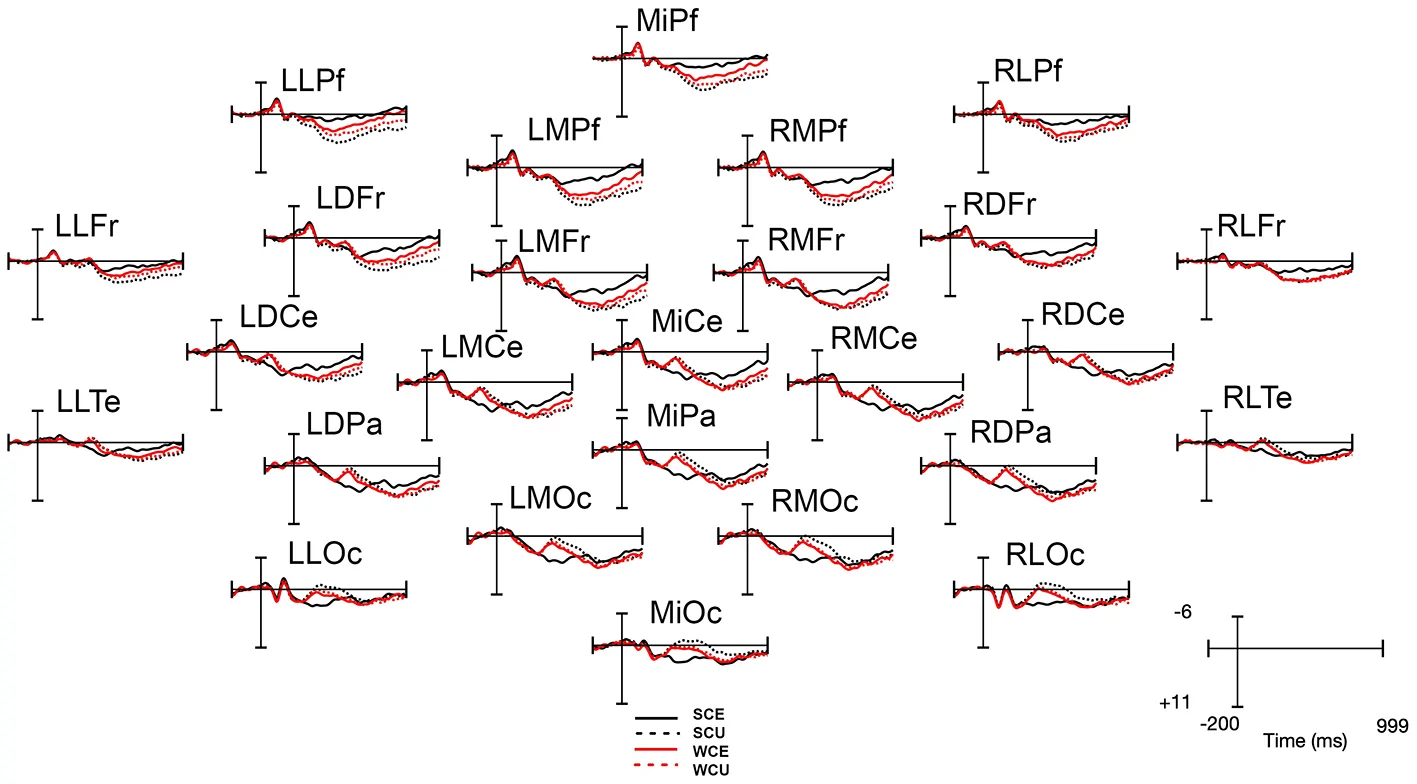
Grand-average ERPs to sentence-final critical words in high-value sentences in Experiment 3. ERPs are time-locked to the onset of the sentence-final word and shown at all scalp electrode sites. Sentence-final words were expected or unexpected completions of strongly constraining sentences (SCE and SCU, respectively) or expected or unexpected completions of weakly constraining sentences (WCE and WCU, respectively). SC, strong constraint; WC, weak constraint; E = expected; U = unexpected. Negative is plotted up.

##### 4.3.2.2 Anterior positivity

To assess the AP, we compared responses to unexpected endings in strongly constraining sentences with responses to unexpected endings in weakly constraining sentences (SCU–WCU). SCU endings elicited more positive responses over frontal electrode sites (600–900 ms) compared to WCU endings, *t*(42) = 3.05, *p* < 0.005, and the effect did not differ by value, *t*(42) = −1.10, *p* = 0.28. This pattern suggests that older adults were engaging prediction mechanisms while reading the sentences, irrespective of sentence value (Figure 19).

**Figure 19:**
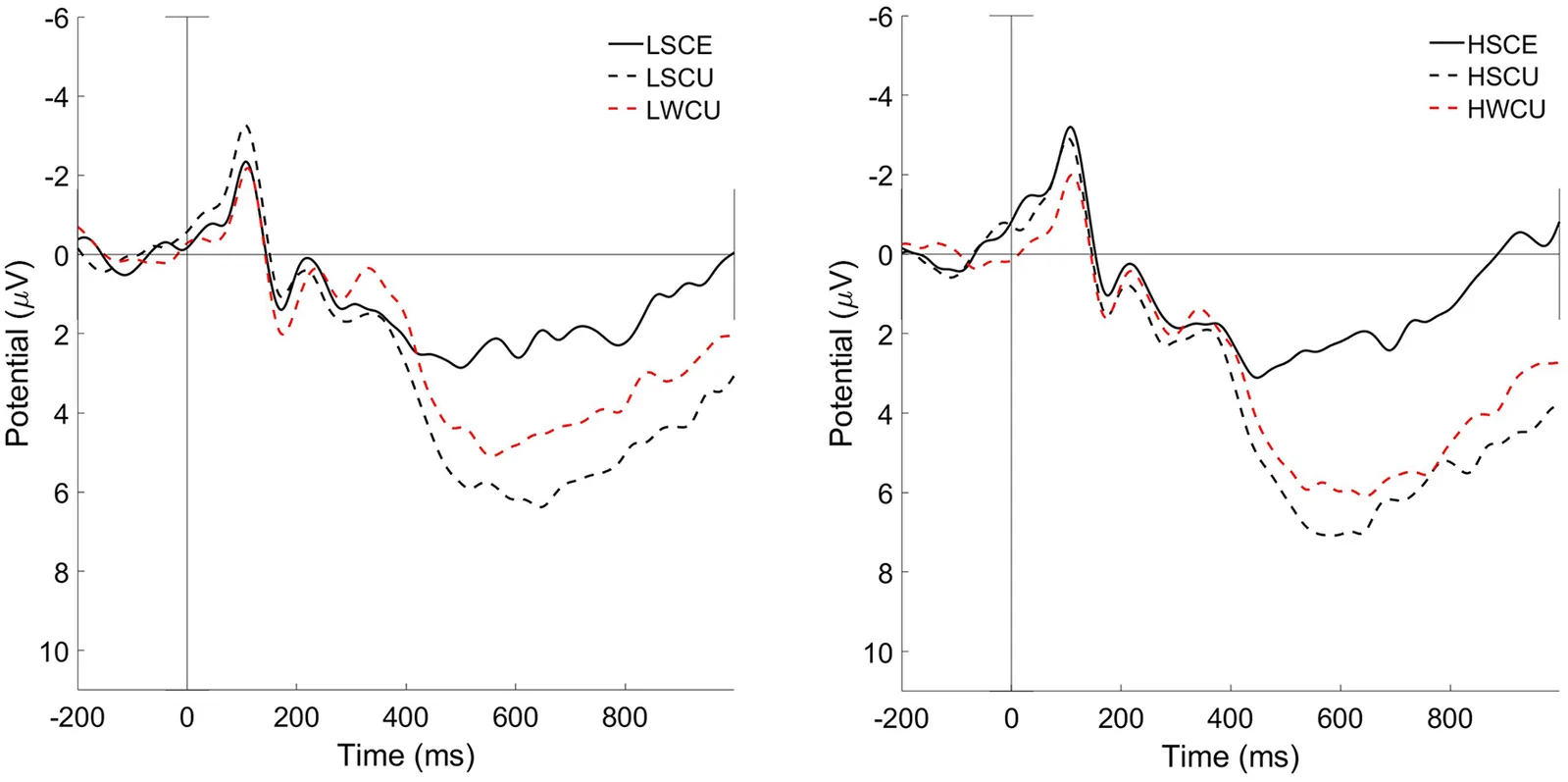
Late prefrontal ERP effects in Experiment 3. Waveforms from representative prefrontal channel LMPf show the contrasts used to isolate the anterior positivity (AP; SCU–WCU) and frontal negativity (FN; SCE–WCU) for low-value sentences **(left)** and high-value sentences **(right)**. Negative is plotted up. An AP effect is evident for both value conditions, with more positive responses to SCU endings. An FN effect (more negative responses to SCE endings) is evident in both value conditions, but larger for high value sentences.

##### 4.3.2.3 Frontal negativity

The FN was assessed using the SCE–WCU contrast, comparing expected completions of strongly constraining sentences with the weakly constraining unexpected baseline. Older adults elicited a reliable FN effect overall, *t*(42) = −5.66, *p* < 0.001. This effect also differed significantly by Value, *t*(42) = −2.13, *p* < 0.05, with high value items eliciting a larger FN (High: Mean = −3.09, SD = 4.00; Low: Mean = −1.60, SD = 3.06); see Figure 19.

##### 4.3.2.4 N400

The N400 expectancy effect was computed as the difference between expected and unexpected sentence-final words, separately for strongly and weakly constraining sentences. Expectancy effects were analyzed using a linear mixed effects model that included Value (High, Low) and Constraint (Strong Constraint, Weak Constraint) and their interaction as fixed effects and a random intercept for participants. Older adults showed greater N400 facilitation in strongly than weakly constraining contexts, β = 1.96, *t* = 4.92, *p* < 0.001, similar to the pattern observed in young adults. However, in contrast to Experiment 1, value did not enhance the N400 expectancy effect in older adults, β = −0.22, *t* = −0.55, *p* = 0.59. The Value × Constraint interaction was not significant, β = 1.37, *t* = 1.72, *p* = 0.09. Thus, older adults showed robust contextual facilitation, but not the value-related enhancement of semantic facilitation observed in younger adults (Figure 20).

**Figure 20:**
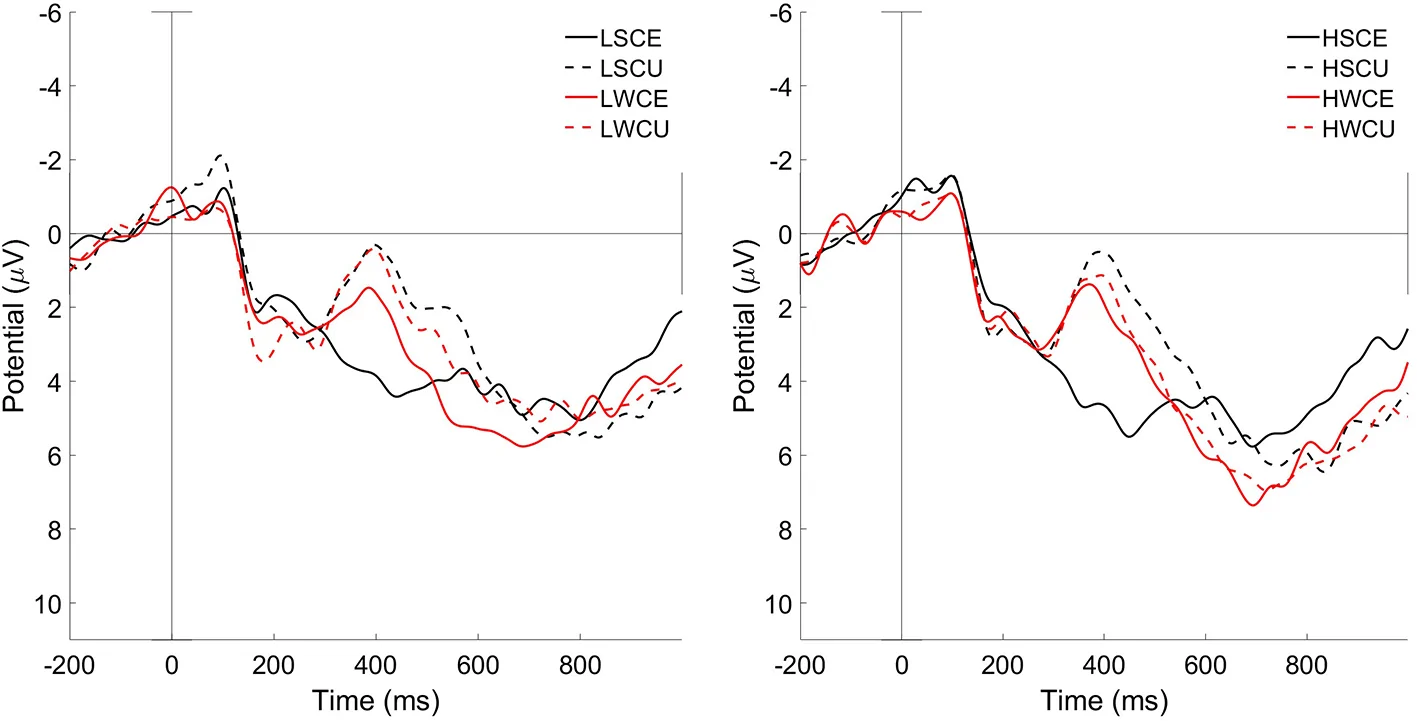
N400 expectancy effects in Experiment 3. Waveforms from representative centroparietal channel MiPa show responses to expected and unexpected sentence-final words in strongly and weakly constraining contexts, separately for low-value sentences **(left)** and high-value sentences **(right)**. SC, strong constraint; WC, weak constraint; E = expected; U = unexpected. Negative is plotted up. Expected words elicited reduced N400s relative to unexpected words, with larger expectancy effects in strongly than weakly constraining contexts, and no effect of value.

##### 4.3.2.5 Trials backsorted by sentence-final word recall

We then asked whether older adults' sentence-final ERP responses were related to later recall of the sentence-final word in context. As in Experiment 1, trials were sorted by Recall Status: trials entered the Recall bin when participants later recalled the sentence together with its sentence-final word or a synonym, and entered the No Recall bin when no associated response was produced. Trials in which participants recalled the gist of the sentence but not the final word were excluded from this ERP backsorting, allowing the analysis to focus specifically on whether processing of the word to which the ERP was time-locked predicted later memory for that word in its sentence context.

For the AP (SCU–WCU), a simple linear model (more complex models would not converge) with fixed effects of Value (High, Low), Recall Status (Recalled, Not recalled), and their interaction revealed significant effects for Value, β = 2.29, *t* = 2.32, *p* < 0.03, and a significant interaction between Value and Recall Status, β = 5.06, *t* = 2.57, *p* < 0.02. For low-value items, trials that elicited a larger AP were more likely to be recalled, whereas this relationship was not observed for high-value items. The main effect of Recall Status was not significant, β = 0.83, *t* = 0.84, *p* = 0.40.

The FN did not show the same recall-related pattern. A linear mixed effects model with Value (High, Low) and Recall Status (Recalled, Not recalled), and their interaction as fixed effects and random intercepts for participants, revealed a significant effect of Value, β = −2.58, *t* = −2.59, *p* < 0.02, but no effect of Recall Status, β = 0.05, *t* = 0.05, *p* = 0.96, nor an interaction, β = −1.89, *t* = −0.95, *p* = 0.34. Similarly, analyses of the N400 expectancy effect (E–U), conducted separately for strongly and weakly constraining sentences, revealed no effects of Recall Status or interactions with Value, all *p* > 0.18. Thus, with the exception of the value-dependent AP relationship, sentence-final ERP measures did not consistently predict later recall.

##### 4.3.2.6 Targeted comparisons with Experiment 1

To directly compare the pattern of effects observed in younger adults (Experiment 1) with older adults (Experiment 3), targeted analyses were conducted for the three ERP components of interest. First, we examined whether there was an overall difference in the effect size of the AP by collapsing across value and comparing between age groups using an independent samples *t*-test. This analysis did not provide evidence that the AP effect differed as a function of age, *t*(89) = 0.50, *p* = 0.62.

Next, for the FN, the impact of value and age was analyzed using a linear mixed effects model that included Value (High, Low) and Age (Younger, Older) and their interaction as fixed effects and a random intercept for participants. High value items elicited a larger FN effect, β = −1.63, *t* = −3.22, *p* < 0.003, and the FN effect was overall larger among older adults, β = −1.90, *t* = −3.59, *p* < 0.001. However, we did not find evidence that the magnitude of value modulation on the FN differs by age group, β = 0.29, *t* = 0.28, *p* = 0.78.

Finally, the N400 expectancy effect was analyzed by calculating each participant's expectancy effect (difference wave) separately for high- and low-value items, collapsed across constraint, and subjected to a linear mixed effects model that included the fixed effects of Value (High, Low), Age Group (Younger, Older), and their interaction. The analysis revealed a significant interaction, β = 1.73, *t* = 2.23, *p* = 0.03, indicating that the effect of value on the N400 expectancy effect differed between younger and older adults. Younger adults exhibited a larger modulation of the N400 expectancy effect by value than did older adults.

##### 4.3.2.7 Summary

The ERP patterns suggest that older adults engaged prediction-related processing during sentence comprehension, as indexed by the presence of an AP to prediction violations. The relationship between this process and subsequent memory varied as a function of value: for low-value items, trials associated with a larger AP were more likely to be recalled, whereas no such relationship was observed for high-value items. At the same time, value influenced later message-level processing, with high-value sentences eliciting a larger FN. In contrast, the N400 expectancy effect varied with constraint but was not modulated by value, indicating that older adults showed contextual facilitation without the value-related enhancement of semantic facilitation observed in younger adults. Overall, these findings suggest that older adults can engage both predictive and integrative mechanisms during comprehension when value information is available in advance, but that value primarily influenced later message-level processing.

Targeted comparisons of the ERP patterns between younger adults and older adults suggest that the influence of aging differs across the three ERP components. The overall AP effect did not differ reliably between younger and older adults, suggesting that both age groups engaged prediction-related processing to a similar degree under these task conditions. Although older adults exhibited a larger FN effect overall, there was no reliable interaction between age group and value, suggesting age-related similarity in how value modulated message-level processing. In contrast, the significant interaction between age group and value observed for the N400 indicates that the influence of value on semantic processing differed between age groups, with younger adults showing a larger value-based modulation of the N400 expectancy effect compared to older adults.

### 4.4 Discussion

The goal of Experiment 3 was to more directly examine older adults' processing strategies when reading sentences associated with different levels of value and to link these processes to subsequent memory. As in the prior experiments, participants read sentences of varying importance and were later tested on recall and recognition; ERPs were recorded to the sentence-final word and also backsorted by recall.

Behaviorally, the most robust and consistent finding across both Experiments 2 and 3 was that older adults used value information to prioritize memory. In particular, older participants consistently showed better recall for high- than low-value sentences, indicating successful value-directed encoding. In contrast, the effect of value on recognition memory differed across experiments: whereas Experiment 2 showed no reliable effect of value, the current study revealed both a main effect of value and an effect of expectedness, driven by reduced recognition for strongly predicted and confirmed endings (SCE items). The emergence of a value effect on recognition may reflect increased task engagement in the in-person setting, as a similar difference was seen for young adults across Experiment 1 and the prior online behavioral study reported in Chung and Federmeier (2023).

Beyond the effect of value, older adults in the current study showed patterns that partially overlapped with those observed in Experiment 2. As in Experiment 2, participants exhibited elevated false alarms to lures relative to new items, consistent with prediction-consistent memory errors. However, false alarms in the current study were reliably greater for strongly constraining lures than weakly constraining lures, indicating a stronger influence of constraint than in Experiment 2 (which showed the same pattern numerically). Responses to hits in both experiments also showed weaker encoding of expected items, with numerically largest effects for strong constraint sentences. In both experiments, older adults' recall was also affected by Constraint (as a main effect in Experiment 3 and an interaction with Expectancy in Experiment 2).

At the neural level, one key question was whether older adults would engage predictive processing under these task conditions. Strikingly, we observed an AP to prediction violations that did not vary with value, indicating that older adults engaged prediction-related revision processes during comprehension. This finding contrasts with prior work showing a lack of AP effects in older adults (Federmeier et al., 2010; Wlotko et al., 2012) and more general findings indicating reductions of engagement in active prediction (DeLong et al., 2012; Federmeier et al., 2002; Federmeier and Kutas, 2019). The comparison with Wlotko et al. (2012) is especially informative because that study used the same sentence materials and found broadly similar N400 and FN patterns, but no AP in older adults reading for comprehension. Thus, the emergence of an AP in the present study points to the task context as an important factor in encouraging predictive processing. To the best of our knowledge, the current study is the first to show older adults, as a group, engaging prediction-related processing without explicit instructions to predict. Prior work has shown that older adults, like younger adults, can exhibit a frontal post-N400 positivity associated with prediction when they are explicitly instructed to generate predictions (Brothers et al., 2017; Dave et al., 2018). In contrast, participants in the current study were free to engage with the sentences however they chose. Their only instructions were to maximize their score by prioritizing high value sentences. These results suggest that older adults can strategically employ predictive processing to complete a memory-demanding task, whereas prior work using standard reading-for-comprehension paradigms have generally found reduced or absent evidence of active prediction in this group. Consistent with this, the direct statistical comparison across age groups showed that the AP effect observed in older adults did not reliably differ from that of younger adults, suggesting that the value-directed memory task elicited prediction-related processing in older adults that was broadly similar to the typical young adult pattern observed in Experiment 1.

Interestingly, this increased effort seems to have a positive impact on older adults' recall for low value items. We found an interaction between recall status and value on the AP effect in which low value items for which older adults engaged in additional revision processes (resulting in a greater difference wave) led to later recall. This pattern was not observed for high value items, toward which older adults were likely more uniformly motivated to allocate effort.

Older adults showed a notable juxtaposition in how this increased engagement with the sentences manifested in the FN and N400. In line with prior literature, older adults showed a FN effect on average (Wlotko et al., 2012). This effect also differed by value in that participants showed a greater FN effect for high value items compared to low value items. This suggests that older adults were selectively and differentially processing sentences as a function of value, potentially engaging in greater levels of message-building for high value items. A direct comparison between younger and older adults found no reliable age-group difference in the value-based modulation of the FN, consistent with the view that value similarly influenced message-level processing in both age groups. At the same time, this value sensitivity did not extend to increased semantic facilitation for high value items for older adults as measured by the expectancy effect on the N400 amplitude. Like in Experiment 1 with younger adults, we observed a greater expectancy effect for strong constraint items compared to weak constraint items but, unlike Experiment 1, we did not observe an effect of value in which high value items experienced greater levels of semantic facilitation. Consistent with this, a comparison between the two age groups revealed an interaction such that younger adults showed a larger value-based modulation of the N400 expectancy effect than older adults. Thus, the overall pattern across components suggests that older adults increase their engagement with high value items as a function of value but that this does not carry over into greater levels of semantic network activation.

Taken together, these findings suggest that older adults can engage predictive and integrative mechanisms during sentence comprehension when motivated by task demands, but that value primarily modulates later stages of message-level integration in ways that are both comparable and different from younger adults. Although some sentence-final ERP measures were related to later memory—notably the interaction between AP, value, and recall status—these relationships were not broad or consistent across components, indicating that, as was also true in Experiment 1, processing at this position alone is not sufficient to fully account for later recall performance.

## 5 General discussion and conclusions

Across three experiments, we investigated whether younger and older adults can use value information to guide their memory for more complex information like sentences, how they may process such information, and what impact those processes may have on later memory of the information. Following Chung and Federmeier (2023), we adapted the value-directed remembering (VDR) paradigm (Castel et al., 2002; Watkins and Bloom, 1999) for use with sentences. Participants were asked to maximize their score after reading unrelated sentences that varied in constraint, predictability and importance. Sentence importance was operationalized by a number value that marked the number of points participants would get for correctly remembering the sentence later on. This value cue appeared either before (Before condition; ERP experiments) or after (After condition) participants read each sentence. Participants completed free recall tasks for the sentences and single-word yes/no recognition tests.

In Experiment 1, we found that young adults elicited a post-N400 AP that was not modulated by value. This effect, operationalized as the difference between equally unexpected items as a function of sentential constraint, provides an index of sensitivity to prediction violation and, hence, a marker of the engagement of active prediction. Consistent with a growing body of prior work, this pattern suggests that young adults engage prediction when reading sentences (see review in Federmeier, 2022) and, here, did so to a similar degree for low and high value items. However, young adults did show signs of selectively increasing engagement with the sentences as a function of value in the FN and N400 expectancy effects. When processing sentences with higher value, young adults elicited larger FN effects and larger N400 expectancy effects, suggesting that participants were engaging in additional message-level processing and showing greater semantic facilitation.

The findings of Experiment 1, taken together with Chung and Federmeier (2023), suggest that young adults as a group can flexibly and differentially engage with study items, both across tasks (as seen in the Before/After comparison in Chung and Federmeier, 2023) and within the same task (as seen in the low/high value items comparison in the current study). They also engage predictive processing during comprehension, which may contribute to the memory distortions observed behaviorally (e.g., increased false recognition for predicted but unseen items and reduced encoding of confirmed predictions), adding to a growing body of evidence that prediction can sometimes be detrimental to memory (e.g., Rommers and Federmeier, 2018a,b; Hubbard et al., 2019; Hubbard and Federmeier, 2024). At the same time, variability in sentence-final processing did not reliably predict later recall, indicating that the processes indexed at this position are not, on their own, sufficient to account for memory outcomes of the full sentence.

In Experiment 2, we examined whether older adults could exert control to remember important but complex information like sentences. Older adults successfully used value to prioritize memory for sentences, replicating the core finding that value-directed remembering extends to more complex, temporally unfolding materials and extending this across the adult life-span. Older adults also showed behavioral signatures consistent with prediction (e.g., lure-based false recognition and reduced memory for prediction-consistent endings). However, these patterns did not vary as a function of when value information was provided, suggesting a reduced flexibility in adapting encoding strategies to task structure compared to younger adults. Because these inferences were based on memory outcomes, Experiment 3 directly assessed online processing.

In Experiment 3, older adults showed an AP to prediction violations that, as in younger adults, was not modulated by value. This finding is striking given that older adults do not typically show such effects during passive comprehension (e.g., Federmeier et al., 2010; Wlotko et al., 2012), and suggests that under task conditions that emphasize memory and provide explicit prioritization cues, older adults can engage prediction even without explicit instructions to predict. This highlights the importance of top-down goals for shaping sentence processing in the moment. Older adults do not seem to engage in active prediction simply when they have to complete a task, whether it is a relatively simple one like answering comprehension questions (DeLong et al., 2012; Wlotko et al., 2012) or a task in which engaging in some level of prediction for upcoming input could be conceivably beneficial, as in a congruency judgement task (Federmeier et al., 2010). Knowing that their memory was going to be tested after the fact also did not lead older adults to spontaneously engage in predictive processing (Federmeier et al., 2002). What may have encouraged older adults in this study to employ more resource intensive processes like prediction is the support that the VDR paradigm provided. In particular, the task provided clear cues (i.e., value) delineating what participants should allocate their resources and efforts toward (i.e., high value items), potentially offsetting age-related deficits in self-initiating strategies (Dunlosky and Hertzog, 2001; Kamp and Zimmer, 2015; Naveh-Benjamin et al., 2007).

Although the task was successful in encouraging prediction in a group that typically does not seem to predict, the findings highlight an important trade-off. Although engaging prediction can increase engagement with the material, it can also distort memory by biasing encoding toward expected information and away from veridical input. Thus, in memory-demanding tasks, prediction may not always be beneficial and may, under some conditions, be counterproductive. Indeed, at least in some cases, providing value information *after* encoding may promote more accurate memory, as people may be less likely to engage in prediction under those conditions. However, there were some selective benefits of prediction for older adults. In particular, in low value items, for which people likely did not engage in extensive rehearsal strategies, predictive processing was linked to better recall of unexpected information. This benefit of revising a prediction violation is reminiscent of how greater resource allocation for conceptual integration at clause or sentence boundaries (i.e., wrap-up) is linked to better memory performance for text-level material (Chin et al., 2015; Stine-Morrow et al., 2001).

Across both age groups, the AP was not modulated by value, adding to a growing body of work showing that this effect is relatively insensitive to task manipulations such as explicit prediction instructions (e.g., Lai et al., 2024) or divided attention (Hubbard and Federmeier, 2021). This pattern suggests that sensitivity to prediction violations, and possibly active prediction processes themselves, may operate in a state-like manner, rather than scaling with effort or strategic prioritization.

In contrast, the most consistent locus of value-based modulation was the FN. This effect, often linked to message-level integration and the resolution of ambiguity, was enhanced for high-value items in both younger and older adults. Notably, the FN is observed even in populations and contexts where prediction is reduced (e.g., Wlotko and Federmeier, 2012a), suggesting that it reflects integrative processes that are distinct from prediction *per se*. Its sensitivity to value aligns with prior work (e.g., Lai et al., 2024) indicating that these later, message-building or refining processes are particularly responsive to task demands and engagement. Backsorting based on recall data did not reveal a direct link between the engagement of these processes and successful recall. However, this may be because the memory benefits that arise from different encoding strategies accrue across the entire sentence and are thus not measurable at the sentence-final word. Moreover, encoding processes alone may not predict recall performance, as memory can also be augmented by post-encoding rehearsal strategies.

The N400 was also sensitive to value, but in a manner that varied with age. Younger adults showed enhanced N400 expectancy effects for high-value items, consistent with prior demonstrations that semantic facilitation can be up- or down-regulated as a function of task demands (e.g., Lai et al., 2024; Hubbard et al., 2019). Older adults, however, showed no modulation of the N400 by value and, more broadly, reduced sensitivity to contextual constraint in weakly constraining sentences, replicating prior findings (Wlotko and Federmeier, 2012b). Prior research suggests that the structure and organization of the semantic network itself is stable in older adults (Jongman and Federmeier, 2022; Payne and Federmeier, 2018; Payne et al., 2015). However, aging may impact how the semantic network is activated and how those activations are adjusted. In particular, typical aging may reduce older adults' ability to rapidly adjust activation states (Jongman and Federmeier, 2022; Payne and Federmeier, 2017a; Wlotko et al., 2012). The absence of additional facilitation for high value items in older adults in the current study may be understood in the same vein: older adults can show signatures of strategic engagement in processes that are brought online relatively later, such as revising prediction violations or enhancing message-level building after encountering the word, but show deficits in adjusting activation states of semantic information, a process that may be more automated (Federmeier, 2022).

Across the behavioral and neural measures, then, the findings suggest that older adults can use value cues, but that the effects of value are more selective than in younger adults. Younger adults showed evidence of modulating their strategies as a function of the timing of value cues (Chung and Federmeier, 2023) and as a function of value within the task on multiple levels, including memory, later message-level processing, and earlier semantic facilitation (Experiment 1). Older adults also used value to support memory and showed value-related modulation of later message-level processing (Experiments 2 and 3). However, they did not show evidence of modulating memory performance depending on when value information became available (Experiment 2), and value did not modulate the N400 in the older-adult ERP sample (Experiment 3). Thus, the present findings are consistent with prior VDR work showing preserved strategic use of value in older adults (Ariel et al., 2015; Castel et al., 2013), while also suggesting that, when value-directed remembering is embedded in real-time sentence comprehension, prioritization goals may not influence all levels of processing equally.

One possibility is that this selectivity reflects the additional demands imposed by processing sentences in real time. Studies of sentence-level and text-level processing have long framed age differences in online comprehension in terms of self-regulated resource allocation: readers must distribute limited processing effort across word-level processing, semantic integration, and discourse-level representation building, and older adults may differ in how flexibly they allocate those resources as comprehension unfolds. In turn, this framing is consistent with broader work suggesting that age-related changes in processes such as attention and inhibitory control are selective and task-dependent rather than global (Rey-Mermet and Gade, 2018; Robertson and Dockree, 2020). Older adults may allocate less time to semantic integration during reading (Miller and Stine-Morrow, 1998; Stine, 1990) despite the memory benefit it can have (Smiler et al., 2003; Stine-Morrow et al., 2010), potentially due to how cognitively demanding wrap-up can be (Payne and Stine-Morrow, 2012, 2014). Older adults also show reduced ability in the moment to use sentential context to facilitate processing of new input (Wlotko and Federmeier, 2012a) or integrate information that has been previously processed (Payne and Federmeier, 2017b).

Several features of the present design should be considered when interpreting the selectivity of the value effects observed here. First, although older adults showed preserved use of value to guide memory and modulate later message-level processing, they did so under a relatively simplified value structure. Prior VDR work using broader value ranges has shown that older adults can show preserved memory for the highest-value items, such as items valued 10–12, while showing age-related reductions for lower-value information or for more specific value representations (Castel et al., 2002, 2007). Thus, although the present design was useful for isolating value effects on online sentence processing, it may not capture the more graded prioritization demands created by broader value ranges. Second, although the present findings point to selective effects of value on different aspects of sentence processing, they do not fully specify why prioritization goals affected later message-level processing but not earlier semantic facilitation in older adults. Using value cues during comprehension likely draws on multiple control processes, including maintaining task goals, allocating attention to high-priority information, and regulating investment in lower-priority information. Age-related changes in attention or inhibitory control could contribute to these patterns, but the present data do not isolate those mechanisms. Future work will be needed to determine when and how strategic prioritization can influence relatively early semantic processing, and how this changes with age. Finally, the older-adult ERP sample was limited by pandemic-related constraints, so replication with a larger sample would be useful, particularly for assessing the selectivity of value effects on the N400 vs. later frontal effects.

In conclusion, we provide evidence that both younger and older adults can use value information to guide memory for complex linguistic input. However, the mechanisms by which value influences processing differ across age. Younger adults flexibly modulate multiple aspects of comprehension, including semantic facilitation, whereas older adults primarily modulate later integrative processes and show reduced flexibility overall. Importantly, with sufficient task support, older adults can engage predictive processing, but this engagement does not fully compensate for age-related changes in more automatic aspects of language comprehension.

## Data Availability

The original contributions presented in the study are publicly available. This data can be found on the Open Science Framework and can be accessed at https://osf.io/yqgmz/.
