# Supplementary material for: Prioritizing meaning: value, prediction, and age differences in sentence processing
Source: Front Cognit. 2026 Oct 8;5:1862899. doi: 10.3389/fcogn.2026.1862899 (PMC13717559; doi:10.3389/fcogn.2026.1862899)
Supplement: Supplementary file 1 [file Supplementary_file_1.docx]

Experiment 1 - Recognition data – False alarms

|  | *Estimate* | SE | *z* | *p* | CI |
| --- | --- | --- | --- | --- | --- |
| Value | -0.04 | 0.10 | -0.43 | 0.67 | [-0.24, 0.16] |
| Category 1 (New vs. Lures) | -0.42 | 0.13 | -3.18 | 0.002 | [-0.67, -0.16] |
| Category 2 (SC Lures vs. WC Lures) | 0.22 | 0.16 | 1.38 | 0.17 | [-0.09, 0.53] |
| Value:Category 1 | 0.02 | 0.12 | 0.20 | 0.84 | [-0.20, 0.25] |
| Value:Category 2 | -0.09 | 0.15 | -0.60 | 0.55 | [-0.37, 0.20] |

Experiment 1 - Recognition data – Hits

|  | *Estimate* | SE | *z* | *p* | CI |
| --- | --- | --- | --- | --- | --- |
| Value | 0.73 | 0.10 | 7.61 | <0.001 | [0.54, 0.92] |
| Constraint | -0.21 | 0.15 | -1.46 | 0.14 | [-0.50, 0.07] |
| Expectedness | -0.23 | 0.15 | -1.57 | 0.12 | [-0.51, 0.06] |
| Value:Constraint | 0.18 | 0.19 | 0.92 | 0.36 | [-0.20, 0.55] |
| Value:Expectedness | -0.01 | 0.19 | -0.03 | 0.98 | [-0.38, 0.37] |
| Constraint:Expectedness | -0.86 | 0.29 | -2.96 | 0.003 | [-1.42, -0.29] |
| Value:Constraint:Expectedness | -0.68 | 0.38 | -1.77 | 0.08 | [-1.43, 0.07] |

Experiment 1 - Recall data

|  | *Estimate* | SE | *z* | *p* | CI |
| --- | --- | --- | --- | --- | --- |
| Value | 1.09 | 0.19 | 5.69 | <0.001 | [0.74, 1.51] |
| Expectedness | -0.16 | 0.10 | -1.65 | 0.10 | [-0.40, 0.01] |
| Value:Expectedness | 0.01 | 0.18 | 0.03 | 0.97 | [-0.33, 0.41] |

Experiment 1 – AP

|  | *Estimate* | *t* | *df* | *p* | CI |
| --- | --- | --- | --- | --- | --- |
| Mu = 0 | 0.67 | 2.02 | 47 | 0.04 | [0.00, 1.33] |

|  | *Estimate* | *t* | *df* | *p* | CI |
| --- | --- | --- | --- | --- | --- |
| Mu (High) – Mu (Low) = 0 | -0.16 | -0.25 | 47 | 0.81 | [-1.46, 1.14] |

Experiment 1 – FN

|  | *Estimate* | *t* | *df* | *p* | CI |
| --- | --- | --- | --- | --- | --- |
| Mu = 0 | -0.44 | -1.30 | 47 | 0.20 | [-1.13, 0.24] |

|  | *Estimate* | *t* | *df* | *P* | CI |
| --- | --- | --- | --- | --- | --- |
| Mu (High) – Mu (Low) = 0 | -1.77 | -2.43 | 47 | 0.03 | [-3.24, -0.31] |

Experiment 1 – N400

|  | *Estimate* | SE | *t* | *P* | CI |
| --- | --- | --- | --- | --- | --- |
| Value | 1.58 | 0.59 | 2.67 | 0.01 | [0.34, -2.69] |
| Constraint | 2.34 | 0.59 | 3.96 | < 0.001 | [1.19, 3.54] |
| Value:Constraint | 1.00 | 1.18 | 0.84 | 0.40 | [-1.29, 3.40] |

Experiment 1 – Anterior Positivity (Back sorted by recall)

|  | *Estimate* | SE | *t* | *p* | CI |
| --- | --- | --- | --- | --- | --- |
| Value | 0.06 | 0.87 | 0.07 | 0.94 | [-1.64, 1.76] |
| Recall | -0.26 | 0.87 | -0.30 | 0.77 | [-1.96, 1.44] |
| Value:Recall | 1.78 | 1.73 | 1.03 | 0.31 | [-1.62, 5.18] |

Experiment 1 – Frontal Negativity (Back sorted by recall)

|  | *Estimate* | SE | *t* | *p* | CI |
| --- | --- | --- | --- | --- | --- |
| Value | 1.29 | 0.92 | 1.40 | 0.16 | [-0.53, 3.11] |
| Recall | 1.31 | 0.92 | 1.42 | 0.16 | [-0.51, 3.14] |
| Value:Recall | -1.59 | 1.85 | -0.86 | 0.39 | [-5.24, 2.05] |

Experiment 1 – N400 for SC sentences (Back sorted by recall)

|  | *Estimate* | SE | *t* | *p* | CI |
| --- | --- | --- | --- | --- | --- |
| Value | 2.80 | 1.02 | 2.76 | 0.007 | [0.71, 4.74] |
| Recall | -0.21 | 1.02 | -0.20 | 0.84 | [-2.19, 1.84] |
| Value:Recall | 2.85 | 2.03 | 1.40 | 0.16 | [-1.30, 6.75] |

Experiment 1 – N400 for WC sentences (Back sorted by recall)

|  | *Estimate* | SE | *t* | *p* | CI |
| --- | --- | --- | --- | --- | --- |
| Value | 1.71 | 1.05 | 1.63 | 0.11 | [-0.54, 3.64] |
| Recall | 0.46 | 1.05 | 0.44 | 0.66 | [-1.52, 2.66] |
| Value:Recall | 0.96 | 2.10 | 0.46 | 0.65 | [-3.42, 4.93] |

Experiment 2 - Recognition data – False alarms

|  | *Estimate* | SE | *z* | *p* | CI |
| --- | --- | --- | --- | --- | --- |
| Condition | 0.43 | 0.28 | 1.54 | 0.12 | [-0.12, 0.99] |
| Category 1 (New vs. Lures) | -0.52 | 0.14 | -3.75 | <0.001 | [-0.79, -0.25] |
| Category 2 (SC Lures vs. WC Lures) | 0.30 | 0.17 | 1.75 | 0.08 | [-0.04, 0.63] |
| Condition:Category 1 | 0.02 | 0.09 | 0.20 | 0.84 | [-0.15, 0.19] |
| Condition:Category 2 | -0.20 | 0.11 | -1.91 | 0.06 | [-0.42, 0.01] |

Experiment 2 - Recognition data – Hits

|  | *Estimate* | SE | *z* | *p* | CI |
| --- | --- | --- | --- | --- | --- |
| Condition | 0.24 | 0.17 | 1.36 | 0.17 | [-0.10, 0.58] |
| Constraint | -0.01 | 0.15 | -0.03 | 0.98 | [-0.30, 0.29] |
| Expectedness | -0.19 | 0.15 | -1.29 | 0.20 | [-0.49, 0.10] |
| Condition:Constraint | -0.03 | 0.14 | -0.23 | 0.82 | [-0.30, 0.24] |
| Condition:Expectedness | 0.13 | 0.14 | 0.95 | 0.34 | [-0.14, 0.40] |
| Constraint:Expectedness | -0.26 | 0.30 | -0.85 | 0.39 | [-0.84, 0.33] |
| Condition:Constraint:Expectedness | -0.61 | 0.28 | -2.18 | 0.03 | [-1.15, -0.06] |

Experiment 2 - Recognition data – Hits (After condition)

|  | *Estimate* | SE | *z* | *p* | CI |
| --- | --- | --- | --- | --- | --- |
| Constraint | 0.01 | 0.17 | 0.04 | 0.97 | [-0.32, 0.33] |
| Expectedness | -0.25 | 0.17 | -1.48 | 0.14 | [-0.58, 0.08] |
| Constraint:Expectedness | 0.04 | 0.34 | 0.12 | 0.91 | [-0.62, 0.70] |

Experiment 2 - Recognition data – Hits (Before condition)

|  | *Estimate* | SE | *z* | *p* | CI |
| --- | --- | --- | --- | --- | --- |
| Constraint | -0.02 | 0.15 | -0.13 | 0.90 | [-0.32, 0.28] |
| Expectedness | -0.14 | 0.15 | -0.89 | 0.37 | [-0.44, 0.17] |
| Constraint:Expectedness | -0.52 | 0.31 | -1.69 | 0.09 | [-1.12, 0.08] |

Experiment 2 - Recall data

|  | *Estimate* | SE | *z* | *p* | CI |
| --- | --- | --- | --- | --- | --- |
| Value | 0.33 | 0.05 | 6.43 | <0.001 | [0.23, 0.43] |
| Expectedness | 0.09 | 0.05 | 1.82 | 0.07 | [-0.01, 0.19] |
| Condition | 0.15 | 0.16 | 0.93 | 0.35 | [-0.17, 0.46] |
| Constraint | 0.39 | 0.13 | 3.09 | 0.002 | [0.14, 0.64] |
| Value:Expectedness | -0.00 | 0.10 | -0.01 | 0.99 | [-0.20, 0.20] |
| Value:Condition | -0.10 | 0.10 | -0.98 | 0.33 | [-0.30, 0.10] |
| Expectedness:Condition | 0.08 | 0.10 | 0.74 | 0.46 | [-0.12, 0.27] |
| Value:Constraint | -0.07 | 0.10 | -0.70 | 0.49 | [-0.27, 0.13] |
| Expectedness:Constraint | 0.49 | 0.10 | 4.80 | <0.001 | [0.29, 0.69] |
| Condition:Constraint | -0.07 | 0.10 | -0.71 | 0.48 | [-0.27, 0.13] |
| Value:Expectedness:Condition | 0.22 | 0.20 | 1.10 | 0.27 | [-0.17, 0.62] |
| Value:Expectedness:Constraint | 0.07 | 0.20 | 0.35 | 0.73 | [-0.33, 0.47] |
| Value:Condition:Constraint | -0.24 | 0.20 | -1.17 | 0.24 | [-0.64, 0.16] |
| Expectedness:Condition:Constraint | 0.03 | 0.20 | 0.15 | 0.88 | [-0.37, 0.43] |
| Value:Expectedness:Condition:Constraint | 0.31 | 0.41 | 0.76 | 0.45 | [-0.49, 1.10] |

Experiment 3 - Recognition data – False alarms

|  | *Estimate* | SE | *z* | *p* | CI |
| --- | --- | --- | --- | --- | --- |
| Value | 0.13 | 0.14 | 0.93 | 0.35 | [-0.15, 0.41] |
| Category 1 (New vs. Lures) | -0.77 | 0.22 | -3.42 | <0.001 | [-1.21, -0.32] |
| Category 2 (SC Lures vs. WC Lures) | 1.09 | 0.37 | 2.98 | 0.003 | [0.37, 1.81] |
| Value:Category 1 | 0.13 | 0.22 | 0.57 | 0.57 | [-0.31, 0.56] |
| Value:Category 2 | -0.80 | 0.37 | -2.14 | 0.03 | [-1.53, -0.07] |

Experiment 3 - Recognition data – Hits

|  | *Estimate* | SE | *z* | *p* | CI |
| --- | --- | --- | --- | --- | --- |
| Value | 0.52 | 0.11 | 4.60 | <0.001 | [0.30, 0.74] |
| Constraint | -0.17 | 0.18 | -0.97 | 0.33 | [-0.52, 0.17] |
| Expectedness | -0.40 | 0.18 | -2.29 | 0.02 | [-0.75, -0.06] |
| Value:Constraint | -0.08 | 0.23 | -0.35 | 0.73 | [-0.52, 0.36] |
| Value:Expectedness | 0.12 | 0.23 | 0.54 | 0.59 | [-0.32, 0.56] |
| Constraint:Expectedness | -0.52 | 0.35 | -1.47 | 0.14 | [-1.21, 0.17] |
| Value:Constraint:Expectedness | -0.54 | 0.45 | -1.20 | 0.23 | [-1.42, 0.35] |

Experiment 3 - Recall data

|  | *Estimate* | SE | *z* | *p* | CI |
| --- | --- | --- | --- | --- | --- |
| Value | 0.73 | 0.11 | 6.58 | <0.001 | [0.51, 0.95] |
| Expectedness | -0.06 | 0.12 | -0.51 | 0.61 | [-0.30, 0.18] |
| Constraint | 0.45 | 0.15 | 3.02 | 0.003 | [0.16, 0.75] |
| Value:Expectedness | -0.02 | 0.16 | -0.15 | 0.88 | [-0.34, 0.29] |
| Value:Constraint | -0.25 | 0.22 | -1.17 | 0.24 | [-0.68, 0.17] |
| Expectedness:Constraint | 0.25 | 0.24 | 1.07 | 0.28 | [-0.21, 0.72] |
| Value:Expectedness:Constraint | 0.01 | 0.32 | 0.02 | 0.98 | [-0.62, 0.64] |

Experiment 3 – AP

|  | *Estimate* | *t* | *df* | *p* | CI |
| --- | --- | --- | --- | --- | --- |
| Mu = 0 | 0.89 | 3.05 | 42 | 0.004 | [0.30, 1.47] |

|  | *Estimate* | *t* | *df* | *p* | CI |
| --- | --- | --- | --- | --- | --- |
| Mu (High) – Mu (Low) = 0 | -0.73 | -1.10 | 42 | 0.28 | [-2.07, 0.61] |

Experiment 3 – FN

|  | *Estimate* | *t* | *df* | *p* | CI |
| --- | --- | --- | --- | --- | --- |
| Mu = 0 | -2.34 | -5.66 | 42 | < 0.001 | [-3.18, -1.51] |

|  | *Estimate* | *t* | *df* | *p* | CI |
| --- | --- | --- | --- | --- | --- |
| Mu (High) – Mu (Low) = 0 | -1.49 | -2.13 | 42 | 0.04 | [-2.90, -0.08] |

Experiment 3 – N400

|  | *Estimate* | SE | *t* | *p* | CI |
| --- | --- | --- | --- | --- | --- |
| Value | -0.22 | 0.40 | -0.55 | 0.59 | [-1.00, 0.56] |
| Constraint | 1.96 | 0.40 | 4.92 | <0.001 | [1.18, 2.73] |
| Value:Constraint | 1.37 | 0.79 | 1.72 | 0.09 | [-0.19, 2.93] |

Experiment 3 – Anterior Positivity (Back sorted by recall)

|  | *Estimate* | SE | *t* | *p* | CI |
| --- | --- | --- | --- | --- | --- |
| Value | 2.29 | 0.98 | 2.32 | 0.02 | [0.34, 4.23] |
| Recall | 0.83 | 0.98 | 0.84 | 0.40 | [-1.12, 2.77] |
| Value:Recall | 5.06 | 1.97 | 2.57 | 0.01 | [1.18, 8.95] |

Experiment 3 – Frontal Negativity (Back sorted by recall)

|  | *Estimate* | SE | *df* | *t* | *p* | CI |
| --- | --- | --- | --- | --- | --- | --- |
| Value | -2.58 | 1.00 | 118.00 | -2.59 | 0.01 | [-4.53, -0.62] |
| Recall | 0.05 | 1.00 | 117.67 | 0.05 | 0.96 | [-1.90, 2.00] |
| Value:Recall | -1.89 | 1.99 | 118.00 | -0.95 | 0.34 | [-5.80, 2.01] |

Experiment 3 – N400 for SC sentences (Back sorted by recall)

|  | *Estimate* | SE | *t* | *p* | CI |
| --- | --- | --- | --- | --- | --- |
| Value | 0.61 | 0.80 | 0.76 | 0.45 | [-0.96, 2.18] |
| Recall | -0.30 | 0.80 | -0.38 | 0.71 | [-1.87, 1.27] |
| Value:Recall | 2.18 | 1.60 | 1.36 | 0.18 | [-0.95, 5.32] |

Experiment 3 – N400 for WC sentences (Back sorted by recall)

|  | *Estimate* | SE | *t* | *p* | CI |
| --- | --- | --- | --- | --- | --- |
| Value | -0.34 | 0.71 | -0.48 | 0.63 | [-1.74, 1.06] |
| Recall | 0.24 | 0.71 | 0.34 | 0.73 | [-1.16, 1.64] |
| Value:Recall | 1.76 | 1.42 | 1.24 | 0.22 | [-1.04, 4.56] |

Experiment 3 – AP (Younger v. Older adults)

|  | *Estimate (Younger)* | *Estimate (Older)* | *t* | *df* | *p* | CI |
| --- | --- | --- | --- | --- | --- | --- |
| Mu (Younger) – Mu (Older) = 0 | 0.67 | 0.89 | 0.50 | 89 | 0.62 | [-0.66, 1.10] |

Experiment 3 – FN (Younger v. Older adults)

|  | *Estimate* | SE | *t* | *p* | CI |
| --- | --- | --- | --- | --- | --- |
| Value | -1.63 | 0.51 | -3.22 | 0.002 | [-2.60, -0.55] |
| Age group | -1.90 | 0.53 | -3.59 | < 0.001 | [-2.96, -0.81] |
| Value:Age group | 0.29 | 1.01 | 0.28 | 0.78 | [-1.80, 2.39] |

Experiment 3 – N400 (Younger v. Older adults)

|  | *Estimate* | SE | *t* | *p* | CI |
| --- | --- | --- | --- | --- | --- |
| Value | 0.65 | 0.39 | 1.67 | 0.10 | [-0.12, 1.41] |
| Age group | 1.65 | 0.39 | 4.25 | < 0.001 | [0.89, 2.41] |
| Value:Age group | 1.73 | 0.78 | 2.23 | 0.03 | [0.20, 3.26] |
